# Comparative genomics analysis of *Stenotrophomonas maltophilia* strains from a community

**DOI:** 10.3389/fcimb.2023.1266295

**Published:** 2023-11-28

**Authors:** Yini Li, Xin Liu, Lingzhi Chen, Xiao Shen, Haihong Wang, Ruiyu Guo, Xiang Li, Zehui Yu, Xiaoli Zhang, Yingshun Zhou, Li Fu

**Affiliations:** ^1^ Department of Ultrasound, Affiliated Hospital of Southwest Medical University, Southwest Medical University, Luzhou, China; ^2^ Department of Pathogen Biology, School of Basic Medicine, Public Center of Experimental Technology of Pathogen Biology Technology Platform, Southwest Medical University, Luzhou, China; ^3^ Laboratory Animal Center, Southwest Medical University, Luzhou, China; ^4^ Department of Allergy, Jiangnan University Medical Center, Wuxi, China; ^5^ Affiliated Hospital of Southwest Medical University, Southwest Medical University, Luzhou, China

**Keywords:** *Stenotrophomonas maltophilia*, whole-genome sequencing, genome analysis, multidrug resistance, biofilm

## Abstract

**Background:**

*Stenotrophomonas maltophilia* is a multidrug-resistant (MDR) opportunistic pathogen with high resistance to most clinically used antimicrobials. The dissemination of MDR *S. maltophilia* and difficult treatment of its infection in clinical settings are global issues.

**Methods:**

To provide more genetic information on *S. maltophilia* and find a better treatment strategy, we isolated five *S. maltophilia*, SMYN41–SMYN45, from a Chinese community that were subjected to antibiotic susceptibility testing, biofilm formation assay, and whole-genome sequencing. Whole-genome sequences were compared with other thirty-seven *S. maltophilia* sequences.

**Results:**

The five *S. maltophilia* strains had similar antibiotic resistance profiles and were resistant to β-lactams, aminoglycosides, and macrolides. They showed similar antimicrobial resistance (AMR) genes, including various efflux pumps, β-lactamase resistance genes (*blaL1/2*), aminoglycoside resistance genes [*aac(6’)*, *aph(3’/6)*], and macrolide-resistant gene (*MacB*). Genome sequencing analysis revealed that SMYN41-SMYN45 belonged to sequence type 925 (ST925), ST926, ST926, ST31, and ST928, respectively, and three new STs were identified (ST925, ST926, and ST928).

**Conclusion:**

This study provides genetic information by comparing genome sequences of several *S. maltophilia* isolates from a community of various origins, with the aim of optimizing empirical antibiotic medication and contributing to worldwide efforts to tackle antibiotic resistance.

## 1 Background

The evolution and dissemination of antibiotic resistance has become a significant threat to public health worldwide, contributing to difficulties in treatment and being associated with high morbidity and mortality (Kaye and Pogue, 2015). *Stenotrophomonas maltophilia* is an emerging multidrug-resistant (MDR) opportunistic human pathogen that often causes nosocomial infections with high resistance to most clinically used antimicrobials (Nicodemo and Paez, 2007; Brooke, 2012; Anđelković et al., 2019; Brooke, 2021; Dadashi et al., 2023). *S. maltophilia* can be found in various environments, from the natural surroundings to the human body, such as the skin, respiratory tract, urinary catheters, and breathing tubes (Denton and Kerr, 1998; Brooke, 2012). Infections generally result in pneumonia, bacteremia, urinary tract infection, or meningitis, particularly those associated with cystic fibrosis or chronic lung disease (Looney et al., 2009; Brooke, 2012). The mortality rate of *S. maltophilia*-related bacteremia ranges from 14% to 69% (Brooke, 2012).

To the best of our knowledge, the major molecular mechanisms of resistance in *S. maltophilia* include intrinsic and acquired antibiotic resistance mechanisms. The major intrinsic resistance mechanism responsible for its MDR phenotype can be attributed to the activity of chromosomally encoded multidrug efflux pumps, low membrane permeability, and antibiotic-modifying enzymes, such as β-lactamases and other aminoglycoside phospho- and acetyl-transferases (Walsh et al., 1997; Looney et al., 2009; Brooke, 2012; Gil-Gil et al., 2020). The genome of *S. maltophilia* encodes various multidrug efflux pumps, including the ATP-binding cassette (ABC)-transporter family, major facilitator superfamily (MFS)-type, resistance nodulation cell division (RND) efflux systems, small multidrug resistance (SMR) family, and fusaric acid resistance efflux pump family (Alonso and Martínez, 2000; Li et al., 2002; Crossman et al., 2008; Al-Hamad et al., 2009; Huang et al., 2013; Lin C-W. et al., 2014; Lin Y-T. et al., 2014). Furthermore, an increasing number of studies have extensively described biofilm formation in *S. maltophilia*, which can lead to infections and antimicrobial resistance (Flores-Treviño et al., 2019). Biofilms are the products of bacterial adherence to natural or living surfaces. The starting point of various infections is often the formation of a biofilm by the infecting organism (Wu et al., 2013). Thus, anti-infective therapy targeting the biofilm phase of an organism is an important for effective treatment.

The treatment of infections caused by *S. maltophilia* is controversial and difficult due to its genotypic and phenotypic variability. Pak et al. reported a case of *S. maltophilia*-associated bacteremia that developed resistance after consecutive treatment with antibiotics. Finally, it is resistant to fluoroquinolones and susceptible to trimethoprim-sulfamethoxazole (SXT) (Pak et al., 2015). However, research has shown that the susceptibility of *S. maltophilia* bacterial isolates to SXT decreased from 97.2% in 2001–2004 to 95.7% in 2013–2016 and varied according to the geographic region (Gales et al., 2019).

The spread of MDR *S. maltophilia* is a global public health concern. Understanding the genetic makeup of such opportunistic pathogens will enable us to optimize antibiotic use for patient treatment and will contribute to the worldwide efforts to tackle antibiotic resistance. Whole-genome sequencing (WGS) is gaining importance in the analysis of bacterial pathogens to provide information on genomic determinants and antimicrobial resistance (AMR) genes. WGS studies allow for comparative genomic analysis of bacterial populations, providing new insights into genetic diversity and evolution. Moreover, global genome-based collections are missing for *S. maltophilia*, which is one of the leading drug-resistant nosocomial pathogens worldwide (Rello et al., 2019; Gröschel et al., 2020; Peykov and Strateva, 2023). Therefore, the present study was conducted with the aim of providing genetic information by comparing the genome sequences of several *S. maltophilia* isolates from a community in China and expecting a better treatment strategy.

## 2 Materials and methods

### 2.1 Bacterial isolation and identification

The strains were isolated from sixty-thirds of sputum or urine samples collected from a community in Luzhou, Sichuan Province, China, in 2017. The inclusion criteria were community, volunteered, consecutive, and normal phenotypes. Samples were plated on TSA medium at 36 °C for 24 h–72 h with vancomycin (16 mg/L) and meropenem (6 mg/L) to select the resistant bacteria. Gram-negative resistant strains were screened using Gram staining and grown at 37°C for 24 h–48 h in lysogeny broth (LB) broth after purifying. Sangon genomic DNA kits were used to extract DNA, amplify, and sequence the 16s rRNA gene sequences. The sequences were compared using the Basic Local Alignment Search Tool (BLAST+ 2.14.0 version, https://blast.ncbi.nlm.nih.gov/Blast.cgi). All procedures were approved by the Regional Committee of Ethics for Human Research of Southwest Medical University.

### 2.2 Antimicrobial susceptibility testing and biofilm formation assay

Antibiotics, including SXT, cefoperazone/sulbactam (SCF), levofloxacin (LVX), norfloxacin (NOR), ciprofloxacin (CIP), minocycline (MIN), ampicillin (AM), gentamicin (GM), cefotaxime (CTX), piperacillin (PIP), aztreonam (ATM), imipenem (IPM), and erythromycin (EM), were determined using Kirby–Bauer disk diffusion interpreted in accordance with the recommendations of the Clinical and Laboratory Standard Institute (CLSI) guidelines (Weinstein and Lewis, 2020). Briefly, MH agar plates were evenly spread over 200 µL of inoculum (0.5 McFarland, 10^8^ CFU/mL) of overnight incubation and dried at room temperature for 5 min. Less than five antibiotic discs (HiMedia Labs) were placed equidistant on each plate. The zones of inhibition (ZOI) were measured using the antibiotic zone scale (Hi-Media Labs) after overnight culture at 37°C according to CLSI (the antimicrobial disk concentration and reference criteria for ZOIs are in **Additional File 1**).

The biofilm formation assay was performed using crystal violet staining. The isolates were cultured overnight in TSB broth for 72 h at 37°C. Aseptic TSB broth was used as the blank control. Each isolate was diluted in fresh TSB broth to achieve a cell density equivalent of 10^8^ CFU/ml. A total of 100 μl of diluted culture was transferred into each microtiter plate (96-well plates, round-bottom) and incubated at 37°C for 72 h. Culture supernatants were discarded and 200 μl of aseptic saline was added to all wells, cleaned three times with distilled water, and plates were allowed to dry at 37°C for 1 h. Then, add 100 μl of 1% crystal violet very well and leave it for 20 min at room temperature to stain the samples for the quantification of biofilms. They were carefully washed them three times with water to remove excess dye, and then dried at room temperature. Finally, dissolve the dye with 100 μl of 30% acetic acid for 30 min. The absorbance of solubilized crystal violet was measured at 595 nm optical density (OD_595_) using 30% acetic acid as a reference. All experiments were performed in triplicate and repeated three times. The optical density cutoff value (ODc) was the average optical density value (OD) of the negative control. The strength of biofilm formation was categorized as follows (Pompilio et al., 2011): no biofilm production (OD ≤ODc), weak biofilm formation (ODc < OD ≤ 2×ODc), moderate biofilm formation (2×ODc < OD ≤ 4×ODc), and strong biofilm formation (OD >4×ODc).

### 2.3 Whole genome sequencing, annotation, and analysis

Genomic DNA of the *S. maltophilia* strains was extracted using a DNA Kit (QIAGEN, Germany). A 300 bp paired-end library was constructed using the standard Illumina DNA sample preparation instructions and then sequenced on MiSeq system sequencing platforms (Novogene, China). Sequence reads were assembled using SPAdes version 3.12 (Lapidus and Korobeynikov, 2021). The whole genome sequence was automatically annotated by the National Center for Biotechnology Information (NCBI) Prokaryote Genome Annotation Pipeline (PGAP) (Li et al., 2021). Functional annotation of genes in the genomes was performed using the Clusters of Orthologous Groups (COGs) (ncbi.nlm.nih.gov/research/cog) and Kyoto Encyclopedia of Genes and Genomes (KEGG) databases (http://www.genome.jp/kegg/). GO annotation of protein-coding genes was performed using BLAST2GO (Conesa et al., 2005; Götz et al., 2008). Antibiotic resistance determinants was annotated using the Resistance Gene Identifier (RGI) in the Comprehensive Antibiotic Resistance Database (RGI 6.0.2, CARD 3.2.7, perfect and strict hits only; card.mcmaster.ca) (Alcock et al., 2023). Acquired resistance genes were predicted using ResFinder 4.1 at the Center for Genomic Epidemiology (CGE, select for 90% threshold and 60% minimal length) (Zankari et al., 2017; Bortolaia et al., 2020). Gene annotations and sequence comparisons were performed using BLAST (query coverage and percentage identity ≥80%) and the DNASTAR software.

### 2.4 Genome data, phylogenetic analysis, and multi-locus sequence typing (MLST)

We researched the available *S. maltophilia* genome sequence data sets from the publicly available genome database NCBI (https://www.ncbi.nlm.nih.gov/), and the high-throughput average nucleotide identity (ANI) analysis of 2.4K prokaryotic genomes reveals clear species boundaries (Richter and Rosselló-Móra, 2009) (**Additional File 2**). We collected the genome sequences of *S. maltophilia* isolates that have highly similar ANI values (>95%) to SMYN41-45 and downloaded several representative isolates from different sources with complete information available. A total of 42 genomic sequences were analyzed in a comparative fashion throughout this study and systematically classified by source, including human, animal, and environmental origin.

For phylogenetic analysis and comparative genome analysis, our isolated *S. maltophilia* strain genome sequences and thirty-seven *S. maltophilia* genome sequences from NCBI were compared using PGAP, with which the genes shared by all genomes were collected, concatenated, and aligned. The phylogenetic tree was constructed using the PanX pipeline (https://pangenome.org/) and was visualized with MEGA11 (Ding et al., 2018; Kumar et al., 2018). PanX is a comprehensive analysis software based on DIAMOND, MCL, and phylogeny-aware post-processing that can identify the core genome and build a strain-level phylogeny using single nucleotide polymorphisms (SNPs) in the core genome in one stop (core genome threshold: default 1.0) (Ding et al., 2018). The core-genome SNP tree was constructed using the script panX.py, which is part of the panX software [code: panX.py -fn./gbk -sl c1 -t 12 -nsl, the input is gbk files are generated from Prokka annotation (https://github.com/tseemann/prokka)] (Seemann, 2014). Software tools were used with default parameters. We clustered strains into clonal groups according to the classification results in studies by Vinuesa, Mercier-Darty, and Gröschel et al. combined with ANI value comparisons of SMYN41–45 (**Additional File 2**) (V et al., 2018; Gröschel et al., 2020; Mercier-Darty et al., 2020). Based on seven housekeeping genes (atpD, gapA, guaA, mutM, nuoD, ppsA, and recA), MLST of whole-genome sequence data of the isolates was performed according to the PubMLST.org website (PubMLST—Public databases for molecular typing and microbial genome diversity) (Jolley et al., 2018). New alleles and sequence types (STs) were confirmed using the University of Oxford database. An MLST minimum spanning tree was constructed using PHYLOViZ Online (PHYLOViZ Online).

### 2.5 Nucleotide sequence accession numbers

The whole genome sequences were deposited in the NCBI database under accession numbers SRZW00000000, SRVN00000000, SRVO00000000, SRVQ00000000, and SRVP00000000 for the *S. maltophilia* strains SMYN41, SMYN42, SMYN43, SMYN44, and SMYN45, respectively.

## 3 Results

### 3.1 The phenotype, antimicrobial susceptibilities, and biofilm formation

Five *S. maltophilia* isolates were identified from the 63 samples collected from the community in China, named SMYN41, SMYN42, SMYN43, SMYN44, and SMYN45, respectively. Almost all five *S. maltophilia* strains were resistant to AM, GM, PIP, CTX, ATM, IPM, and EM, whereas they were almost susceptible to SXT, SCF, LVX, NOR, CIP, and MIN according to CLSI breakpoints (M100-S23) (**Table 1**, at the end of this text).

**Table 1:** Antibiotic susceptibility and the optical density value of biofilm in *Stenotrophomonas maltophilia* SMYN41–45.

Antibiotics	SXT	SCF	LVX	NOR	CIP	MIN	AM	GM	PIP	CTX	ATM	IPM	EM	OD *	biofilm classification
**SMYN41**	S	S	S	S	S	S	R	R	R	I	I	R	R	0.101	weak
**SMYN42**	S	S	S	S	I	S	R	R	R	I	I	R	R	0.123	moderate
**SMYN43**	S	S	S	S	S	S	R	R	I	R	R	R	R	0.115	moderate
**SMYN44**	S	I	S	S	S	S	R	R	R	R	R	R	R	0.218	strong
**SMYN45**	S	I	S	S	S	S	R	R	R	R	R	R	R	0.170	moderate
Susceptibility rate	100%	86%	100%	100%	86%	100%	0	0	0	0	0	0	0	NA	NA
Intermediary rate	0	14%	0	0	14%	0	0	0	14%	29%	29%	0	0	NA	NA
Resistance rate	0	0	0	0	0	0	100%	100%	86%	71%	71%	100%	100%	NA	NA

*: the average optical density (OD) value of biofilm formation of S. maltophilia SMYN41-45 and the mean value of the negative control wells is 0.051.

SXT, trimethoprim/sulfamethoxazole; SCF, cefoperazone/sulbactam; LVX, levofloxacin; NOR, norfloxacin; CIP, ciprofloxacin; MIN, minocycline; AM, ampicillin; GM, gentamicin; PIP, piperacillin; CTX, cefotaxime; ATM, aztreonam; IPM, imipenem; EM, Erythromycin; S: susceptibility; R: Resistance; I: Intermediary; NA: Not applicable.

The OD_595_ values of the biofilm-formation assays are shown in **Table 1**. Isolate SMYN41 could formed weak biofilm (OD_595_: 0.101; ODc = 0.051), while isolates SMYN42, SMYN43, and SMYN45 have produced moderate biofilm (OD_595_: 0.115–0.170). Isolate SMYN44 is a strong biofilm-producer (OD_595_: 0.218).

### 3.2 Characteristics of the whole genome

The general genomic features of the five isolates sequenced in this study are summarized in **Additional File 3**. Lengths of whole genome sequences of strain SMYN41–SMYN45 have total sizes ranging from 4,371,193 to 4,897,474 bp with no plasmid. The genomes consist of 72, 18, 17, 62, and 32 contigs with a G + C content of 66.60%, 66.72%, 66.31%, 66.72%, and 66.59%, respectively. The predicted genes were annotated using the COG, KEGG, and GO gene databases (**Additional Files 4**-**18**). Specifically, a total of 3,070 (67.94%), 2,828 (72.36%), 2,828 (72.46%), 2,986 (70.31%), and 2,938 (71.78%) genes that were functionally annotated according to GO were classified into three categories (biological process, cellular component, and molecular function). A total of 3,825 (84.64%), 3,419 (87.49%), 3,419 (87.60%), 3,608 (84.95%), and 35.47 (86.66%) genes belonging to 24 categories were annotated from the COG database. Based on searches against the KEGG database, 1,952 (43.20%), 1,865 (47.72%), 1,863 (47.73%), 1,983 (44.57%), and 1,886 (46.08%) genes were predicted.

### 3.3 Biofilm-forming relative genes

**Figure 1:**
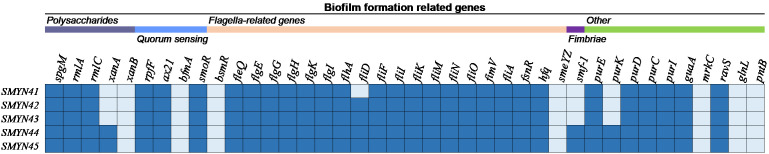
Genes associated with biofilm formation in the five *S. maltophilia* strains. The isolates are shown on the left, and the biofilm-forming genes are shown at the top. Dark blue color indicates the presence of the gene, and light blue indicates its absence.

There are 40 genes associated with different mechanisms of biofilm formation in *S. maltophilia* SMYN41–SMYN45 that are annotated based on the NCBI PGAP (**Figure 1**). All these genes were located on the chromosome. Genes responsible for polysaccharide production (*spgM*, *rmlA*, and *rmlC*) (Huang et al., 2006; Pompilio et al., 2011; Zhuo et al., 2014; Madi et al., 2016), quorum sensing (QS) (*rpfF*, *ax21*, and *smoR*) (Bahar et al., 2014; Devos et al., 2015; García et al., 2015; Huedo et al., 2015; Martínez et al., 2015; Ryan et al., 2015; Huedo et al., 2018), and flagella (*fleQ*, *flgE/G/G/K/I*, *flhA, fliF/I/K/M/N/O/A*, and *fimV*) (Roscetto et al., 2012; Yang et al., 2014; Kang et al., 2015; Liu et al., 2017; Kim et al., 2018) were identified in *S. maltophilia* SMYN41–SMYN45 (Flores-Treviño et al., 2019). Several other biofilm-related genes (*purE/D/C/I*, *guaA*, and *ravS*) were annotated in the five strains (Kang et al., 2015). Moreover, the *fliD* gene was annotated in SMYN42–SMYN45 (Kang et al., 2015). SMYN44 and SMYN45 contain the fimbriae gene *smf-1* (Gallo et al., 2016), polysaccharide production gene *xanA* (Kang et al., 2015), and other *purK* genes (Kang et al., 2015).

### 3.4 Antimicrobial resistance analysis

**Figure 2:**
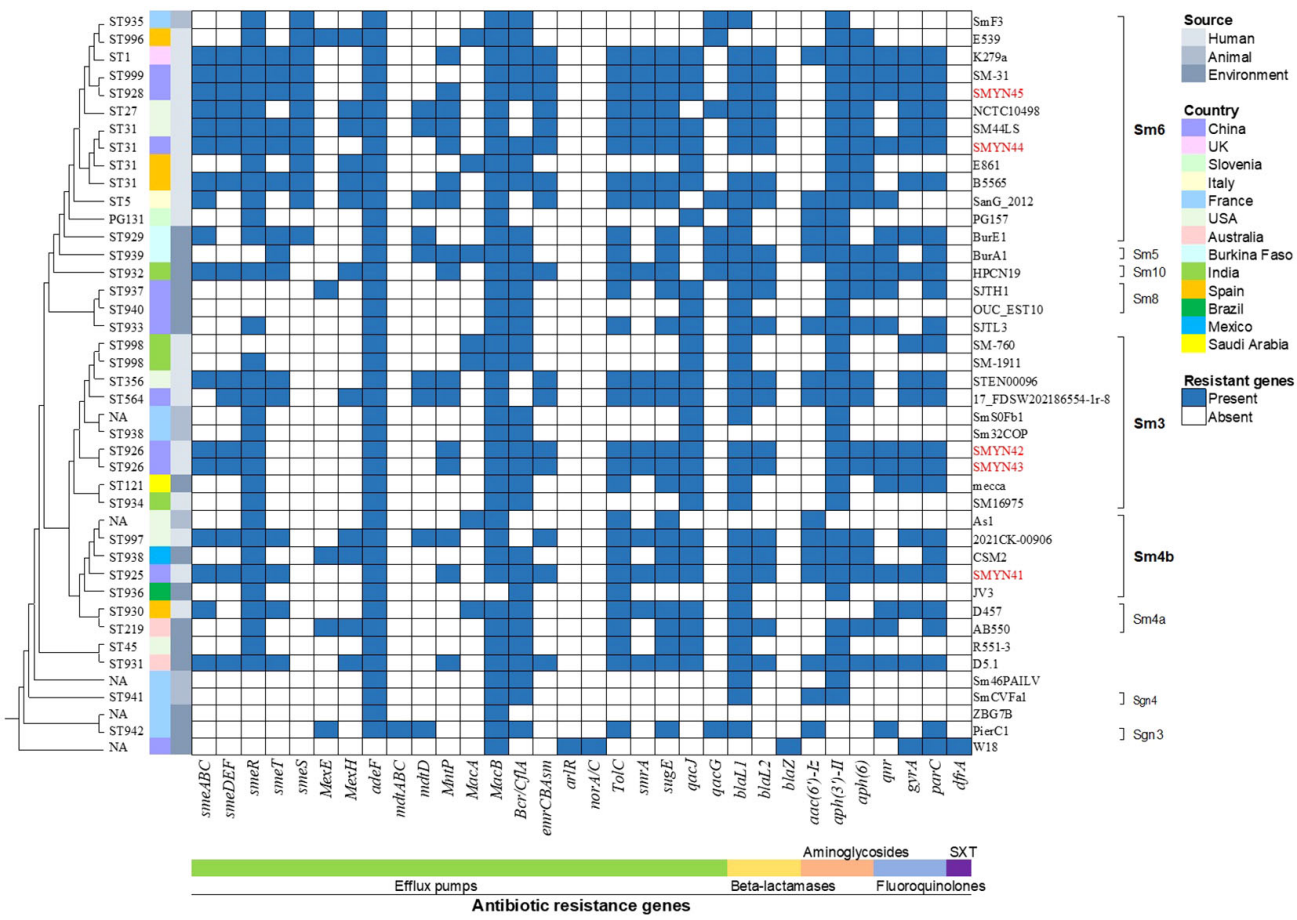
Phylogenetic tree and antimicrobial resistance genes in 30 *Stenotrophomonas maltophilia* strains. Isolates and lineages are shown on the right. The sequence types (STs) are shown on the left (NA, not assigned). The resistance genes are shown at the bottom of the figure.

A total of 34 genes involved in different mechanisms of drug resistance were annotated and identified based on the NCBI PGAP and CARD (**Figure 2**). All these genes were located on the chromosome. Several genes encoding the RND family (*smeABC*, *smeDEF*, *adeF*, *MntP*, and *MacB*), SMR family (*qacJ*), and MFS efflux pumps (*bcr/CflA*, *emrCBAsm*, and *TolC* protein families) were identified in SMYN41–SMYN45. The five genome sequences also contained a variety of AMR genes, including those conferring aminoglycoside resistance (*aph(3’)-II* and *aph(6)*), β-lactam resistance (*blaL1* and *blaL2*), macrolide resistance (*MacB*), and fluoroquinolone resistance (*qnr*, *gyrA*, and *parC*). The presence of point mutations in *gyrA* and *parC* may be responsible for fluoroquinolone resistance in strains. However, we did not find point mutations in *gyrA* or *parC*. Furthermore, SMYN44 and SMYN45 contain *smeS*, and the aminoglycoside resistance gene *aac(3’)-Iz* was identified in SMYN41. Notably, aminoglycoside resistance genes [*aph(3’)-IIc* and *aac(6’)-Iz*], as well as β-lactamase blaL1, were confirmed as acquired resistance genes based on ResFinder. However, they cannot be detected by CARD, and a possible reason may be that these acquired AMR gene hits have an unknown phenotype and unknown PubMed Unique Identifier. However, the SXT resistance-related genes (*sul1*, *sul2*, and *dfrA*) were not present in these five genomes.

The AMR gene profiles of SMYN41–SMYN45 were compared with those of 32 *S. maltophilia* isolates obtained from the NCBI database (**Figure 2**). Based on the origin of the strains, *S. maltophilia* SMYN41–SMYN45 conferred similar AMR genes in all human-derived strains. SMYN41 shares almost identical antibiotic genes with *S. maltophilia* K279a, and *S. maltophilia* SMYN44–SMYN45 possessed similar antibiotic genes. Moreover, isolates of animal origin contained the fewest AMR genes and did not contain SXT- and fluoroquinolone-related resistance genes. Environment-derived strains contain different AMR genes and fewer genes than strains of human origin. Overall, 38 of 42 *S. maltophilia* isolates conferred three or more classes of antibiotic-resistant genes. Thirty-four isolates harbored *sme* efflux pumps. Most *S. maltophilia* isolates (36/42) belonged to the SMR family (*qacJ* or *qacG*) associated with resistance to disinfecting agents and antiseptics. Almost all strains contained the macrolide resistance gene *macB*, except for *S. maltophilia* JV3. Beta-lactam resistance genes (38/42) and aminoglycoside resistance genes (39/42) were present in most of these 42 *S. maltophilia* isolates. Isolates As1, E539, E861, Sm32COP, and ZBG7B did not confer beta-lactam resistance, and aminoglycoside resistance genes were not identified in D457, W18, and ZBG7B. Seventeen isolates conferred the quinolone resistance genes *qnr* and *gyrA*/*parC*. However, no mutations were detected in *gyrA* or *parC* genes. Only *S. maltophilia* W18 contained MFS efflux pumps *arlR* and *norA/C*, β-lactamase *blaZ*, and SXT resistance gene *dfrA*, while none of other genomes conferred.

### 3.5 Phylogenetic analysis

We have downloaded the complete sequences of 37 *S. maltophilia* strains from the NCBI database, and the characteristics are summarized in **Table 2**. These strains were isolated from humans (n = 16), animals (n = 6), or the environment (n = 15). The complete genomes of these sequences have total sizes ranging from 4,065,399 to 5,192,275 bp. The antibiotic phenotypes of most of these strains have not yet been described in the literature.

**Table 2:** General features of 5 new *Stenotrophomonas maltophilia* strains and 32 *S. maltophilia* isolates available in NCBI.

Stenotrophomonas maltophilia strain	source	location	Genome size (bp)	Accession no.	Antibiotic phenotype
Human origin					
**17_FDSW202186554-1r-8**	hospital patient	China: Beijing (2020)	4,488,050	JAEDWG000000000	Unknown
**2021CK-00906**	Homo sapiens	USA (2021)	5,192,275	ABLOMS000000000	Unknown
**B5565**	bronchitis	Spain: Barcelona (2011)	4,955,091	RAUR00000000	Resistant to AMK and COL
**D457**	Clinical	Spain: Mostoles	4,769,156	NC_017671	Resistant to TET, ERY, NAL, NOR, and OFX
**E539**	Pus from a wound	Spain: Madrid (1993)	4,608,247	NEQZ00000000	Resistant to CS, CAZ and PM
**E861**	sputum	Spain: Madrid (1994)	4,721,817	NERB00000000	Resistant to CAZ, PM, CS, IMI, and ETP
**K279a**	blood	UK: Bristol (2010)	4,851,126	NC_010943	Multi-drug
**NCTC10498**	hospital patient	USA: Maine (2017)	4,648,315	JACJGP000000000	Unknown
**PG 157**	perineum	Slovenia: Golnik (2011)	4,949,420	LNIW00000000	Unknown
**SanG_2012**	lung	Italy: Rome (2012)	4,909,273	NZ_MQZU00000000	Unknown
**SM-16975**	blood	India (2012)	4,582,432	NZ_LXXZ00000000	Unknown
**SM-1911**	Pus	India (2010)	4,270,469	LXXQ00000000	Unknown
**SM-31**	Homo sapiens	China: Hefei (2017)	4,829,247	JAAAFS000000000	Unknown
**SM44LS**	hospital patient	USA: San Diego (2020)	4,771,078	DAOKHL000000000	Unknown
**SMYN41**	urine	China: Sichuan (2017)	4,897,474	NZ_SRZW00000000	Multi-drug
**SMYN42**	urine	China: Sichuan (2017)	4,371,421	NZ_SRVN00000000	Multi-drug
**SMYN43**	urine	China: Sichuan (2017)	4,545,272	NZ_SRVO00000000	Multi-drug
**SMYN44**	urine	China: Sichuan (2017)	4,666,132	NZ_SRVQ00000000	Multi-drug
**SMYN45**	sputum	China: Sichuan (2017)	4,371,193	NZ_SRVP00000000	Multi-drug
**STEN00096**	sputum	USA: Pittsburgh (2019)	4,333,398	JADUKZ000000000	Unknown
Animal origin					
**As1**	mosquito body	USA (2012)	4,460,063	LFKU00000000	Unknown
**Sm32COP**	horse manure	France: Feucherolles (2010)	4,548,960	LYVH00000000	Susceptible
**Sm46PAILV**	horse manure	France: Feucherolles (2010)	4,123,397	NZ_LYVJ00000000	Unknown
**SmCVFa1**	cattle manure	France: Versailleux (2012)	4,264,176	LZPD01000000	Unknown
**SmF3**	cattle manure	France: Feucherolles (2007)	4,595,297	LYVK01000000	Unknown
**SmSOFb1**	horse manure	France: Saint Olive (2012)	4,483,386	LZPC00000000	Unknown
Environmental origin					
**AB550**	water	Australia, Perth (2016)	4,943,426	CP028899	Unknown
**BurA1**	soil	Burkina Faso (2008)	4,360,660	CVIW00000000	Multi-drug
**BurE1**	soil	Burkina Faso (2008)	4,504,590	CVIU00000000	Multi-drug
**CSM2**	laboratory sink	Mexico: Morelos (2016)	4,739,049	CP025298	Unknown
**D5.1**	mining waste	Australia: Donnybrook (2016)	4,626,840	SRHZ00000000	Multi-drug
**HPCN19**	sewage	India: Nagpur (2016)	4,738,774	QGKQ01000000	Unknown
**JV3**	plant	Brazil (2013)	4,544,477	NC_015947	Unknown
**Mecca**	public	Saudi Arabia (2015)	4,386,843	CWHS00000000	Unknown
**OUC_Est10**	soil	China: Qingdao (2013)	4,668,743	CP015612	Unknown
**PierC1**	soil	France (2015)	4,638,575	CVIV00000000	Susceptible
**R551-3**	poplar tree	USA: Washington (2011)	4,573,969	NC_011071.1	Susceptible
**SJTH1**	wastewater	China: Shanghai (2017)	4,932,325	CP027562	Unknown
**SJTL3**	wastewater	China: Shanghai (2017)	4,891,004	CP029773	Unknown
**W18**	soil	China: Tianjin (2012)	4,738,432	NZ_CP028358	Unknown
**ZBG7B**	soil	France: Zellenberg (2014)	4,065,399	NZ_JXIP01000000	Unknown

Unknown, Strains for which the antibiotic resistance profile is not described in the references.

AMK, amikacin; COL, colistin; TET, tetracycline; ERY, erythromycin; NAL, nalidixic acid; NOR, norfloxacin; OFX, ofloxacin; CS, colistin; CAZ, ceftazidime; PM, cefepime; IMI, imipenem; ETP, ertapenem.

The phylogenetic tree is shown in **Figure 2** and **Additional File 19**. Most strains were not grouped within clusters based on their origins. The environmentally derived strain CSM2 from the Mexican laboratory sink clustered with the human strain SMYN41 from China. The human-derived strains SMYN42 and SMYN43 clustered with animal origin strains Sm32COP and SmSOFb1, environmental origin isolate Mecca, and human-derived isolate SM16975. Moreover, strain k297a from human origin and BurE1 from the Burkina environment clustered with SMYN45 from Chinese human origin and SmF3 from French cattle manure. These results confirm that the phylogeny does not cluster these strains based on their geography and origin (human, environmental, or animal origin).

Similarly, this phylogeny was not grouped within clusters by the multidrug-resistant and antibiotic-susceptible strains. Despite the lack of information for many sequenced strains, according to the known drug-resistant phenotype, for instance, the MDR strains k297a, BurE1, and BurA1, and susceptible strains Sm32COP, R551-3, and PierC1 seem to belong to different clusters. Although E539 is genetically similar to k297a, it cannot be confirmed that MDR strains are grouped within clusters. The resistance gene profiles may not be related to the resistant phenotype. For example, MDR strain E861 contained fewer resistance genes than the susceptible strain B5565 (**Table 2**).

The results of the known genogroup classification are shown in **Figure 2**. Genogroup Sm6 was the most common lineage (36%), with 84.6% of isolates from humans (11/13), which included isolates SMYN44 and SMYN45. Lineage Sm3 also possessed a high proportion of anthropogenic strains (6/10) with SMYN42 and SMYN43. Isolate SMYN41 belongs to lineage Sm4b. Genogroups Sgn3 and Sgn4 are the most distantly related lineages.

### 3.6 MLST results

Based on the housekeeping genes under the Oxford MLST scheme, we searched and submitted the sequences of these 42 strains on pubMLST (**Figure 2** and **Additional File 20**). MLST sequencing showed that *S. maltophilia* SMYN42, SMYN43 and SMYN45 were new STs, and SMYN41–SMYN45 belonged to ST325, ST926, ST926, ST31, and ST928, respectively. Isolates SMYN42 and SMYN43 possess the same housekeeping gene alleles, and both belong to ST926. Among these 32 sequences downloaded from the NCBI database, 20 types are the new STs (ST929–ST942 and ST996–ST999). Twelve isolates consisted of existing types in the database and four (SMYN44, B5565, E861, and SM44LS) belonged to ST31. We could not find completely trusted alleles or hits in the other five isolates, including As1, Sm46PAILV, SmSOFb1, W18, and ZBG7B. The MLST minimum spanning tree further confirmed that this phylogeny is not grouped within clusters by source or antibiotic resistance (**Additional Files 21**, **22**).

## 4 Discussion

In the present study, we characterized the whole-genome sequencing features of five human-origin *S. maltophilia* MDR isolates SMYN41-SMYN45 from a community and performed comparative analysis of biofilm-forming genes in the five isolates as well as AMR genes in a total of 42 *S. maltophilia* isolates.

Based on the antibiotic susceptibility test, it can be concluded that *S. maltophilia* SMYN41–SMYN45 isolated from a community were MDR bacteria resistant to aminoglycosides, carbapenems, macrolides, and glycopeptide antibiotics. The phenotypic antibiotic susceptibility results were consistent with the antibiotic resistance predicted by AMR genes. Furthermore, multidrug resistance was preserved throughout the *S. maltophilia* strains, with most isolates (38/42) harboring multiple AMR genes predicted to be resistant to three or more similar classes of antibiotics. This confirms that starins of different origins share similar resistance determinants (Mercier-Darty et al., 2020). However, environmental strains tended to contain fewer resistance genes than human-associated strains, which was consistent with the findings of Gröschel et al. (2020). Notable, it has been reported that the resistance of *S. maltophilia* to quinolones may be mainly caused by mutations at the target sites of DNA gyrase and topoisomerase (mainly *gyrA* and *parC*), plasmid or chromosome-mediated mutations of drug resistance genes (such as *Qnr* family), and drug efflux pumps (mainly *smeDEF*, which is the major determinant of quinolone resistance in *S. maltophilia*) (Drlica and Zhao, 1997; Alonso and Martinez, 2001; Sanchez et al., 2004; García-León et al., 2014; Jia et al., 2015). SMYN41–SMYN45 conferred the latter two mechanisms, but were not resistant to quinolones. This phenomenon is consistent with that reported by Youenou et al. (2015). The possible reasons are unknown gene expression and that *S. maltophilia* contains a chromosomally encoded *qnr* gene that confers low-level resistance to quinolones upon its expression in a heterologous host (Shimizu et al., 2008; Sánchez and Martínez, 2010). Resistance to various antibiotics limits the choice of therapeutic drug. Moreover, several studies have reported that antibiotic resistance of *S. maltophilia* in clinical settings is associated with previous antibiotic treatment (Wang et al., 2017; Ibn Saied et al., 2020; Yang et al., 2021). Therefore, although these *S. maltophilia* isolates were susceptible to quinolones at that time, the predicted quinolone resistance genes may indicate possible resistance to quinolones after antibiotic use in the future. Additionally, the use of quinolones may lead to SXT resistance (Wang et al., 2017). Multidrug-resistant *S. maltophilia* strains SMYN41–SMYN45 from the community are susceptible to SXT and do not possess SXT-related resistance genes. Studies have found that fluoroquinolone-containing regimens may be a better option than SXT-containing regimens in treating *S. maltophilia*-related infections (Mojica et al., 2016; Lai et al., 2023). Therefore, the treatment of choice for *S. maltophilia* infections remains unclear when the strain is susceptible to both SXT and quinolones.

In addition to the inherent resistance genes of *S. maltophilia*, biofilm formation also exhibits greater resistance to antimicrobial drugs, which is an important virulence feature. Biofilms are difficult to treat clinically (Wang et al., 2016). SMYN41–SMYN45 all can form biofilms. Among several major classes of genes known to be associated with biofilm formation, weak biofilm-producing SMYN41 possesses the fewest biofilm-associated genes. Among them, the crucial role played by bacterial flagella in biofilm development has been well recognized (Guttenplan and Kearns, 2013). Inactivation of some flagellar genes, such as *filA* and the orphan response regulator *FsnR*, can result in deficiencies in biofilm formation (Gallo et al., 2016). These genes are present in SMYN41–SMYN45 and can contribute to biofilm formation. Furthermore, it has been shown that almost all harbored *smf-1* isolates can form biofilm (Gallo et al., 2016). However, our results show that *smf-1* may not be indispensable for biofilm production. There could be a strong correlation between the results of the ability of biofilm production and biofilm-forming genes. Of course, regulatory processes and variations in the expression of genes are significant; therefore, these need to be further verified (Mercier-Darty et al., 2020). Additionally, according to the antimicrobial susceptibility test results, the strong biofilm-producing SMYN44 and SMYN45 strains with the same biofilm-forming gene cluster showed relatively high antibiotic resistance. Notably, the use of fluoroquinolones in the early stages of *S. maltophilia* infection may inhibit biofilm formation (Wang et al., 2016). However, previous studies have suggested that the use of quinolones may lead to subsequent SXT resistance. Therefore, the timing of quinolone use remains controversial. In hospital-acquired infections, the increased *S. maltophilia*-related infections are mainly due to inadequate use of antibiotics, which may also be further complicated by biofilm production (Looney, 2005). Therefore, the use of biofilm-based antibiotic susceptibility testing to select antimicrobials for *S. maltophilia* infections may provide an accurate and effective guide for appropriate therapeutic drug selection (Wu et al., 2013).

The five isolates from the community had a high degree of similarity in COG, KEGG, and GO classifications and similar KEGG pathways, indicating that genes related to essential processes were mostly conserved in evolution for the human and environmental settings. According to the phylogenetic results, there was no obvious evolutionary correlation between the phenotypic profiles and their origins. However, human-derived isolates are prone to contain more antibiotic resistance genes. These results are consistent with those of previous studies (Valdezate et al., 2004; Zhao et al., 2015; Xiao et al., 2021). In line with previous reports, we agree that genogroup Sm6 consists mostly of human derived *S. maltophilia* strains, as well as genogroup Sgn2 and Sgn4 are the more distantly placed lineages (Patil et al., 2018; Gröschel et al., 2020; Mercier-Darty et al., 2020; Yero et al., 2020). Strains with ANI comparisons above 95% belonged to the same lineage. We found that the phylogenetic clades of bacteria SMYN44, SMYN45, and K279a, which all belong to group Sm6, are very close to each other and have strikingly similar antibiotic-resistant genotypes. However, SMYN41 homologs of CSM2, both belonging to lineage Sm4b, have widely varying drug-resistant genotypes. This also exists among many strains. Interestingly, isolates SM44LS, SMYN44, E861, and B5565, with the same sequence type 31, contained different AMR genes. As demonstrated by Zhao et al., small differences in nucleic acid levels could lead to various significant phenotypes in strains with close genetic relationships (Zhao et al., 2015). These results underline the importance of both genetic diversity and conservation.

According to the whole-genome MLST results, one of the most practical sequence types validating databases, three new STs (ST925, ST926, and ST928), and one existing ST were identified from our primary *S. maltophilia* isolates SMYN41–SMYN45, which are similar in AMR and biofilm-forming gene profiles (Kozyreva et al., 2017). Isolates SMYN42 and SMYN43 were the same sequence types, which indicated the possible clonal transmission of *S. maltophilia* infections in the community. The new STs in this study indicate that the isolates from the community in China were different from those from other sources. The sequence types of the total of 37 isolates were quite scattered, indicating loose associations. The phylogenetic tree by pan-genome analysis and the MLST-spanning tree demonstrated that *S. maltophilia* is not clustered by isolation source or geographical origin, which is consistent with many relevant studies (Nicoletti et al., 2011; Youenou et al., 2015; Duan et al., 2020; Yero et al., 2020). However, the MLST spanning tree was not consistent with the phylogenetic tree, which is not in agreement with the results of Gröschel et al. (2020). The possible reason for this could be that insufficient strains were included. More community-sourced isolations must be included to confirm this hypothesis further.

In conclusion, *S. maltophilia* strains isolated from the community are MDR strains that play fundamental roles in hospital-acquired infections, with high resistance to most clinically used antibiotics. They are susceptible to quinolones and SXT antibiotics with relatively conserved gene expression but do not possess the resistance gene of the latter. The different isolates possessed similar AMR gene classes. The strong adhesion of the biofilm also confirmed that it is highly susceptible to infections and difficult to treat clinically. It has been shown that antibiotic treatment is also responsible for its acquisition of resistance. These data support the current treatment regimens (SXT or fluoroquinolones) and suggest the risk of nosocomial infection with *S. maltophilia* (Farrell et al., 2010; Cho et al., 2014; Watson et al., 2018; Ko et al., 2019). However, more data are required. Furthermore, the relevance and risk of MDR transmission among humans, animals, and the environment should also be considered, although phenotypic profiles, genomes, origins, and geographical features cannot discriminate against *S. maltophilia* isolates. Continuous monitoring of *S. maltophilia* strains is needed and may be able to identify variations in the antibiotic resistant phenotypes and AMR genes, and further reveal a high risk of this MDR pathogen.

## 5 Conclusions

Overall, understanding the genetic determinants, biofilm-forming genes, and AMR genes of these human-derived *S. maltophilia* strains will help optimize empirical antibiotic medication, improve the surveillance and prevention of hospital outbreaks of *S. maltophilia*, and contribute to the global implementation of more effective infection prevention and control strategies. Our *in vitro* results provide more meaningful genetic information and an experimental basis for a possible optimal treatment strategy for *S. maltophilia* infections.

## Data availability statement

The datasets presented in this study can be found in online repositories. The names of the repository/repositories and accession number(s) can be found in the article/**Supplementary Material**.

## Ethics statement

The studies involving humans were approved by Ethics Committee of Southwest Medical University. The studies were conducted in accordance with the local legislation and institutional requirements. The participants provided their written informed consent to participate in this study.

## Author contributions

YL: Supervision, Writing – original draft, Writing – review & editing, Data curation, Formal Analysis, Investigation, Methodology, Project administration, Software. YZ: Supervision, Writing – original draft, Writing – review & editing, Resources, Validation. XinL: Formal Analysis, Investigation, Methodology, Writing – review & editing. LC: Data curation, Formal Analysis, Investigation, Methodology, Writing – original draft. XS: Investigation, Methodology, Project administration, Writing – review & editing. HW: Formal Analysis, Investigation, Methodology, Resources, Writing – review & editing. RG: Conceptualization, Investigation, Project administration, Software, Writing – review & editing. XiaL: Formal Analysis, Investigation, Methodology, Writing – review & editing. XZ: Resources, Supervision, Writing – review & editing, Funding acquisition. LF: Resources, Supervision, Writing – review & editing, Software. ZY: Methodology, Writing – review & editing.

## Glossary

MDR: multidrug resistant
MFS: major facilitator superfamily
SMR: small multidrug resistance
SXT: trimethoprim-sulfamethoxazole
WGS: whole-genome sequencing
AMR: antimicrobial resistance
SCF: cefoperazone/sulbactam
LVX: levofloxacin
NOR: norfloxacin
CIP: ciprofloxacin
MIN: minocycline
AM: ampicillin
GM: gentamicin
CTX: cefotaxime
PIP: piperacillin
ATM: aztreonam
IPM: imipenem
EM: erythromycin
BLAST: Basic Local Alignment Search Tool
CLSI: Clinical and Laboratory Standard Institute
NCBI: National Center for Biotechnology Information
PGAP: Prokaryote Genome Annotation Pipeline
COG: Clusters of Orthologous Group
KEGG: Kyoto Encyclopedia of Genes and Genomes
RGI: Resistance Gene Identifier
SNP: Single-nucleotide polymorphism
MCA: multiple correspondence analysis
AMK: amikacin
COL: colistin
CS: colistin
CAZ: ceftazidime
PM: cefepime
IMI: imipenem
ETP: ertapenem
