# Supplementary material for: Comparative genomics analysis of Stenotrophomonas maltophilia strains from a community
Source: Front Cell Infect Microbiol. 2023 Nov 28;13:1266295. doi: 10.3389/fcimb.2023.1266295 (PMC10715271; doi:10.3389/fcimb.2023.1266295)

Additional files

Additional file 1.

Antimicrobials disk concentration and reference criteria of the inhibition zones according to the Clinical and Laboratory Standard Institute (CLSI) guidelines.

Antimicrobial Agent	Disk Content	Zone Diameter Interpretive Criteria (mm)			QC strain*
		S	I	R	
**Sulfamethoxazole/trimethoprim**	1.25/23.75μg	≥16	11~15	≤10	24~32
**Cefoperazone/sulbactam**	10/10μg	≥21	16~20	≤15	19~24
**Levofloxacin**	5μg	≥17	14~16	≤13	29~37
**Norfloxacin**	10μg	≥17	13~16	≤12	28~35
**Ciprofloxacin**	5μg	≥21	16~20	≤15	30~40
**Minocycline**	30μg	≥19	15~18	≤14	15~25
**Ampicillin**	10μg	≥17	14~16	≤13	16~22
**Gentamicin**	10μg	≥15	13~14	≤12	16~26
**Cefotaxime**	30μg	≥23	15~22	≤14	15~32
**Aztreonam**	100μg	≥21	18~20	≤17	28~36
**Imipenem**	10μg	≥23	20~22	≤19	26~32
**Erythromycin**	15μg	≥23	17~22	≤16	22~34

* QC strain: Quality control strain *Escherichia coli* ATCC® 25922
Abbreviations: S: susceptibility; I: Intermediary; R: Resistance.

Additional file 3
The general genomic features of *Stenotrophomonas maltophilia* SMYN41-45

Sample	Reads num	rRNA/tRNA /ncRNA*	GC (%)*	Total sequence length	ORF number*	Contigs num	Scaffolds num	CRISPRs num	Plasmid num	Accession No.
**SMYN41**	11,245,150	4/63/46	66.60	4,897,474	4,519	72	48	9	0	NZ_SRZW00000000
**SMYN42**	11,230,088	3/62/35	66.72	4,371,421	3,908	18	14	3	0	NZ_SRVN00000000
**SMYN43**	12,070,774	3/64/43	66.72	4,371,193	3,903	17	12	2	0	NZ_SRVQ00000000
**SMYN44**	11,527,924	3/64/39	66.31	4,666,132	4,247	62	56	11	0	NZ_SRVO00000000
**SMYN45**	11,639,194	3/62/30	66.59	4,545,272	4,093	32	23	12	0	NZ_SRVP00000000

* GC (%): GC content
* ORF number: The number of open reading frame
* rRNA/tRNA/ncRNA: The number of rRNA (5s, 16s, 23s), tRNA and ncRNA

Additional file 4

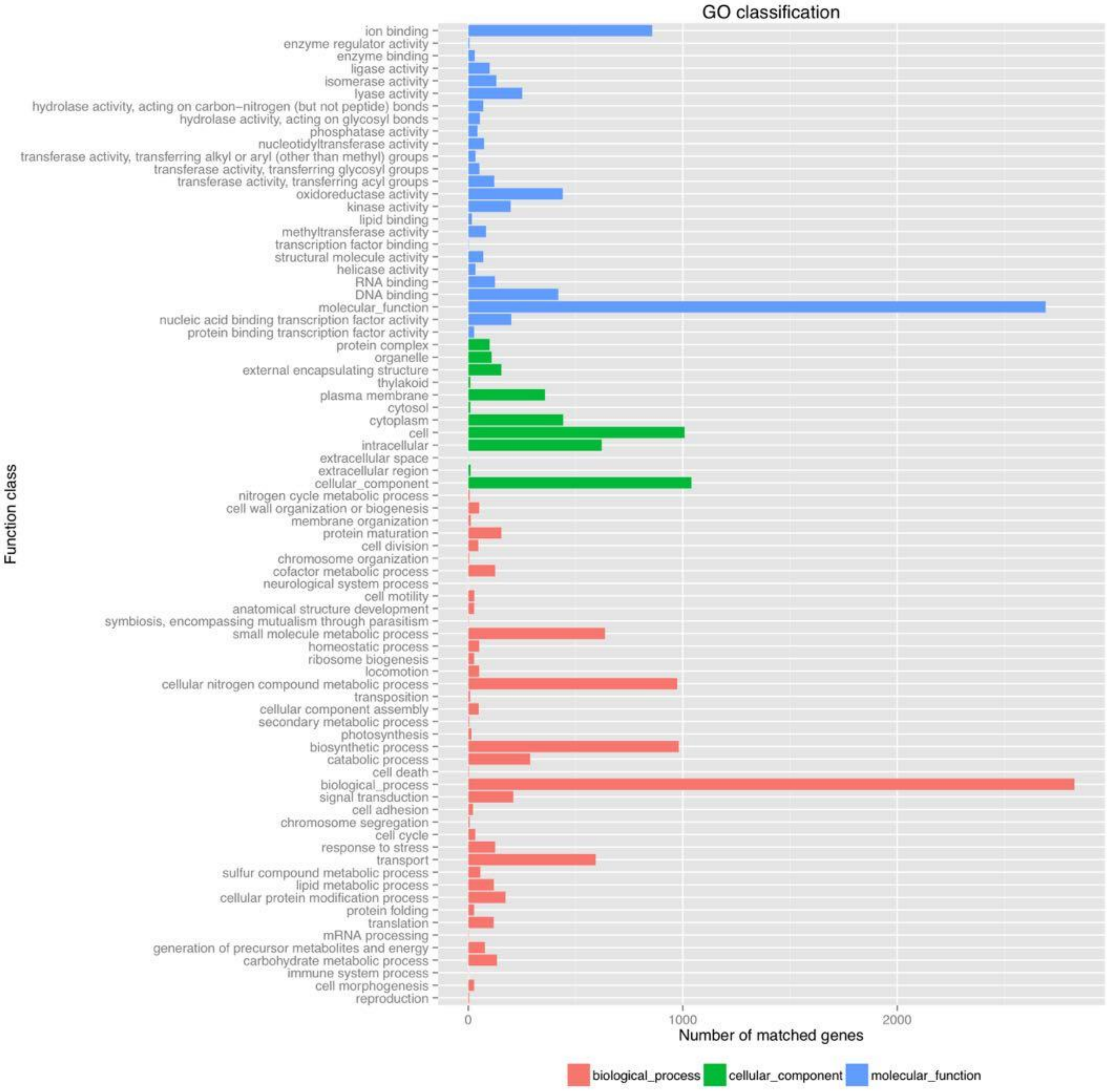

Additional file 5

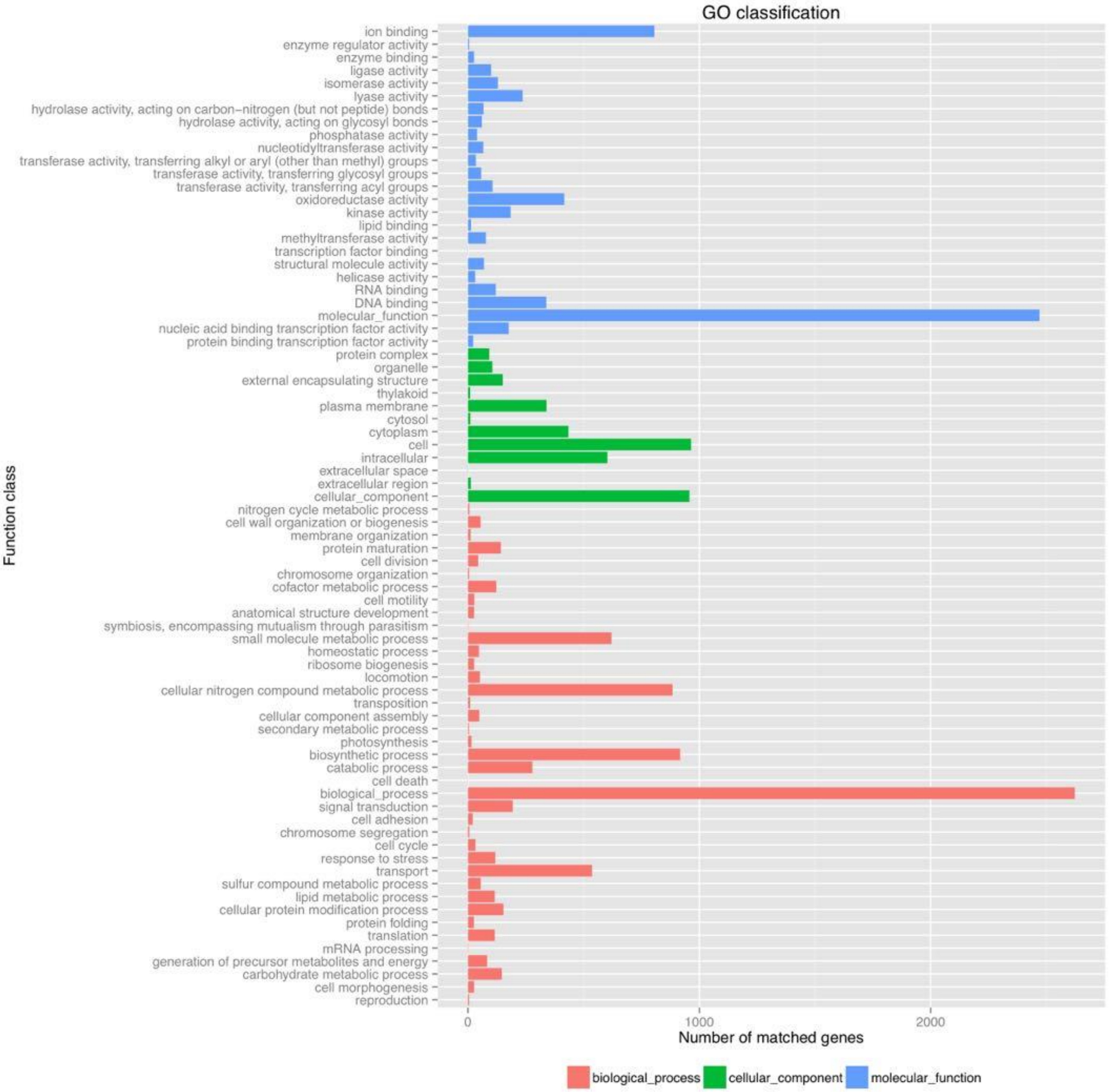

Additional file 6

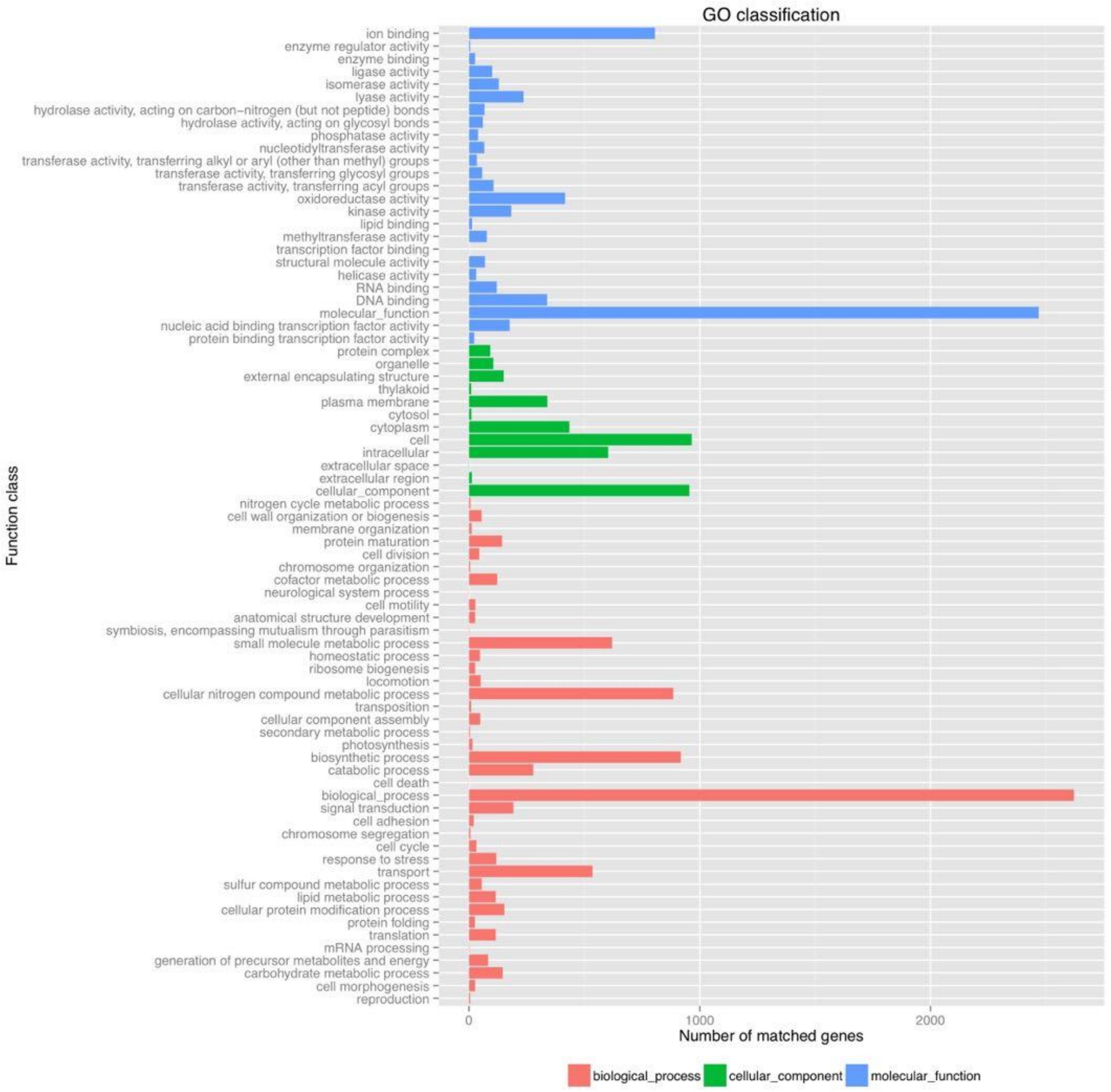

Additional file 7

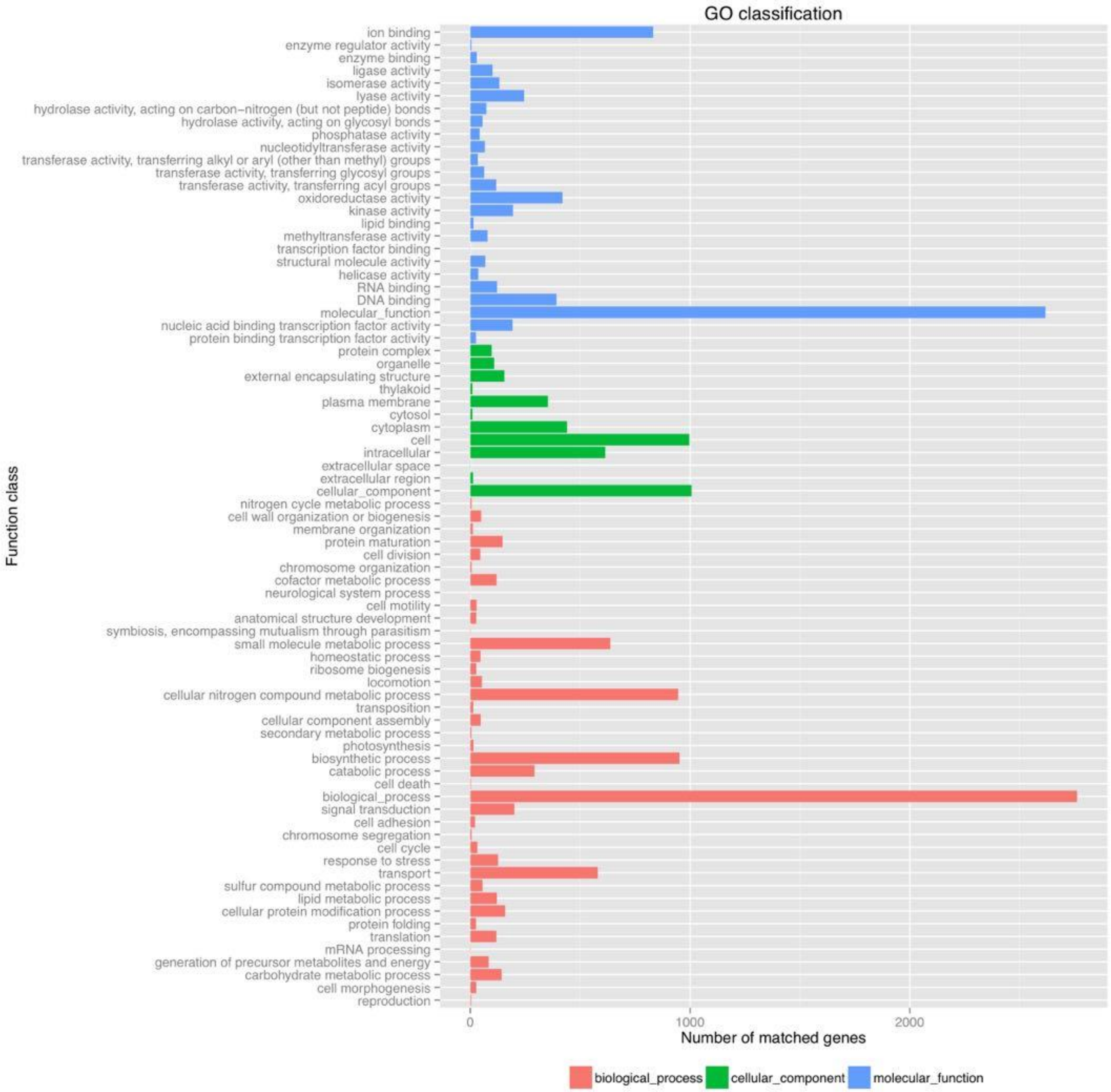

Additional file 8

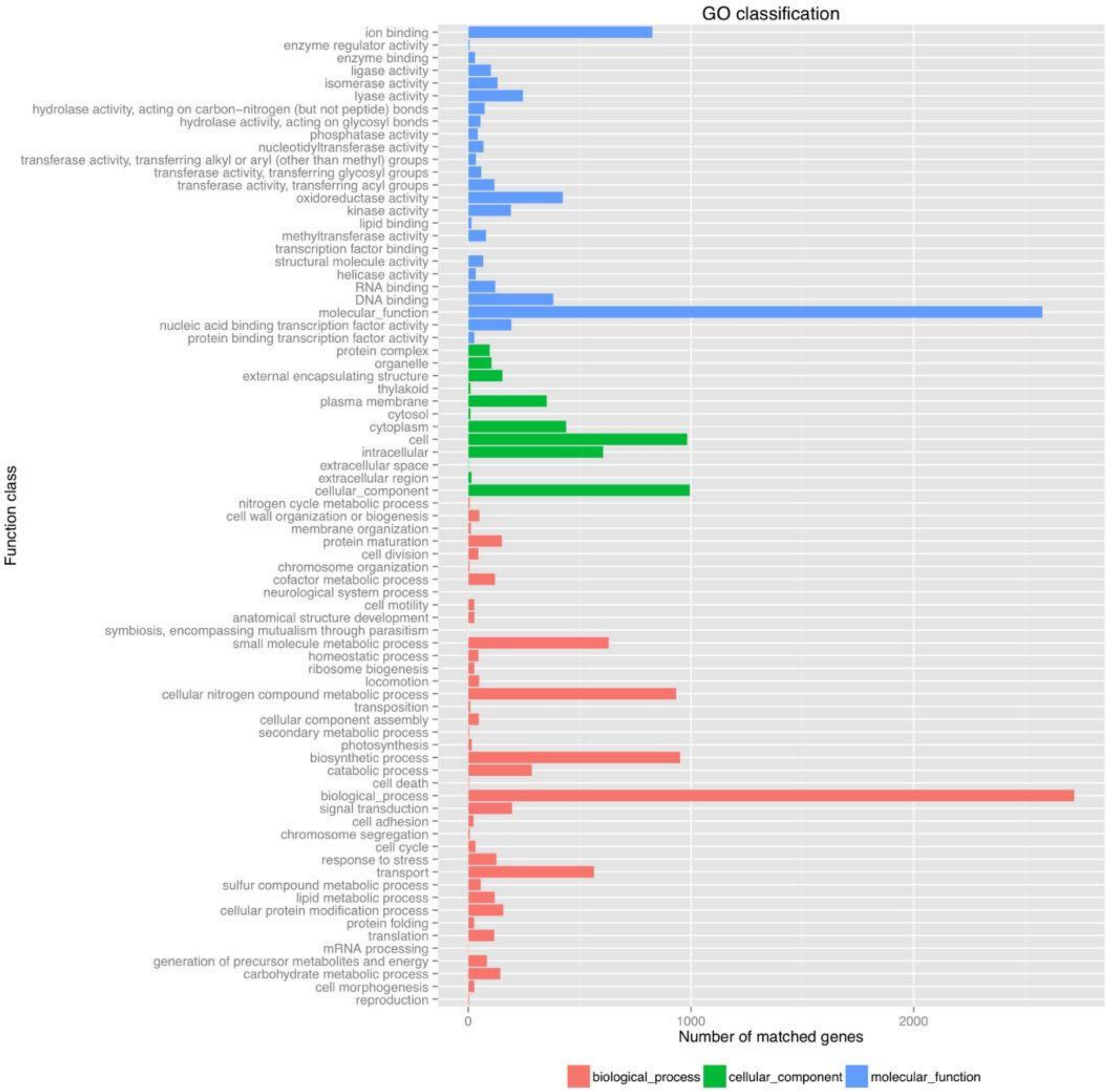

Additional file 9

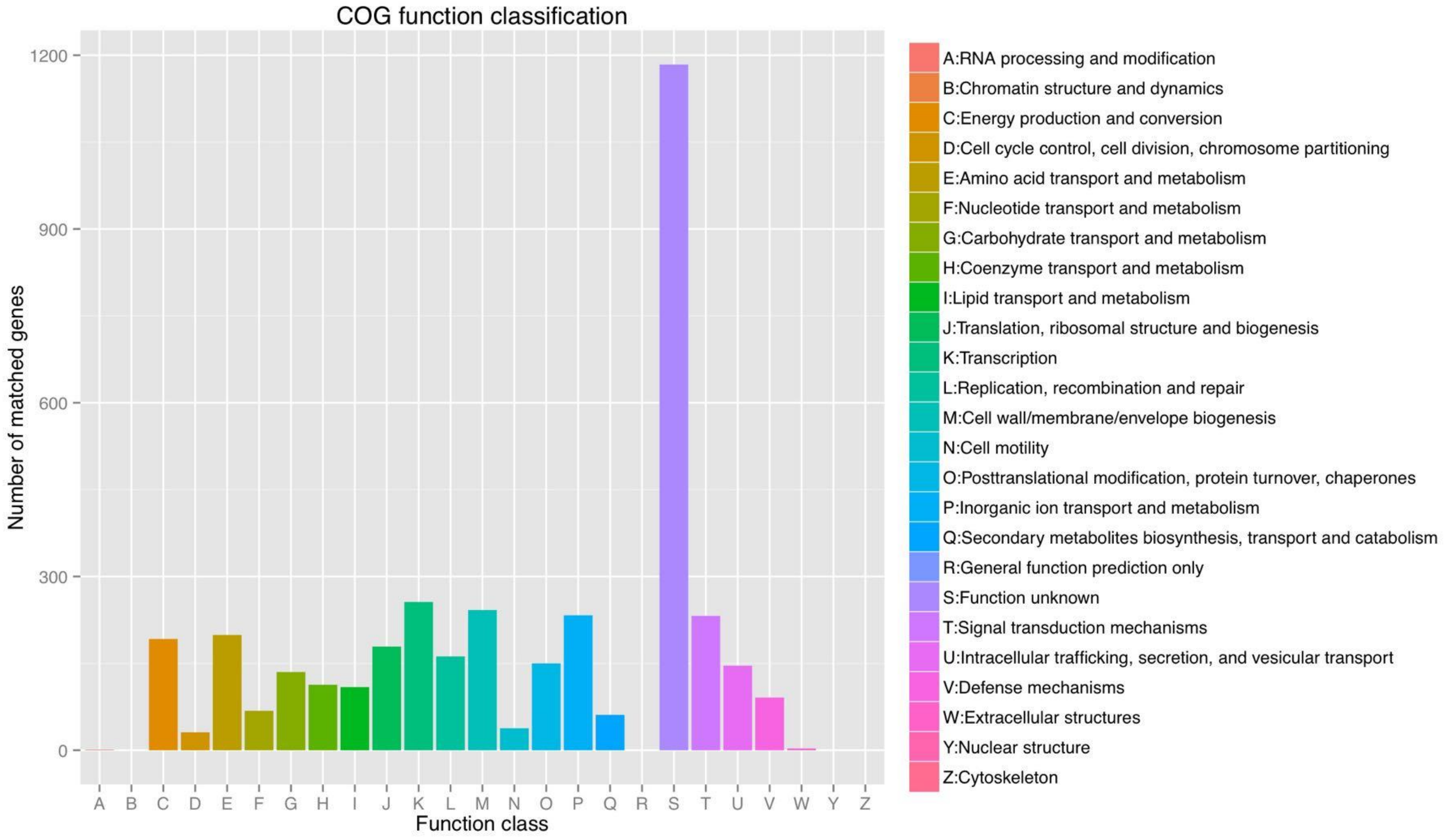

Additional file 10

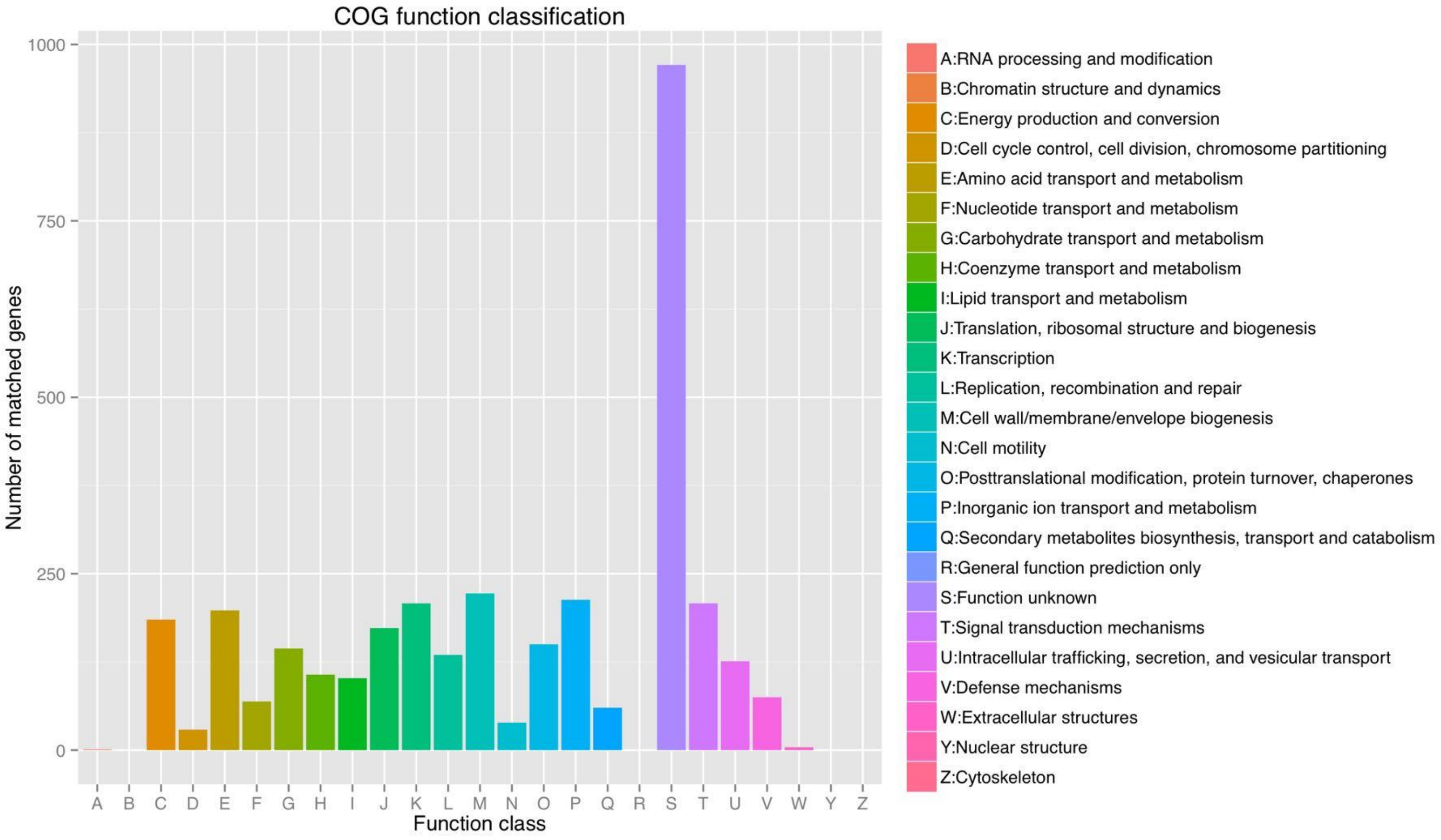

Additional file 11

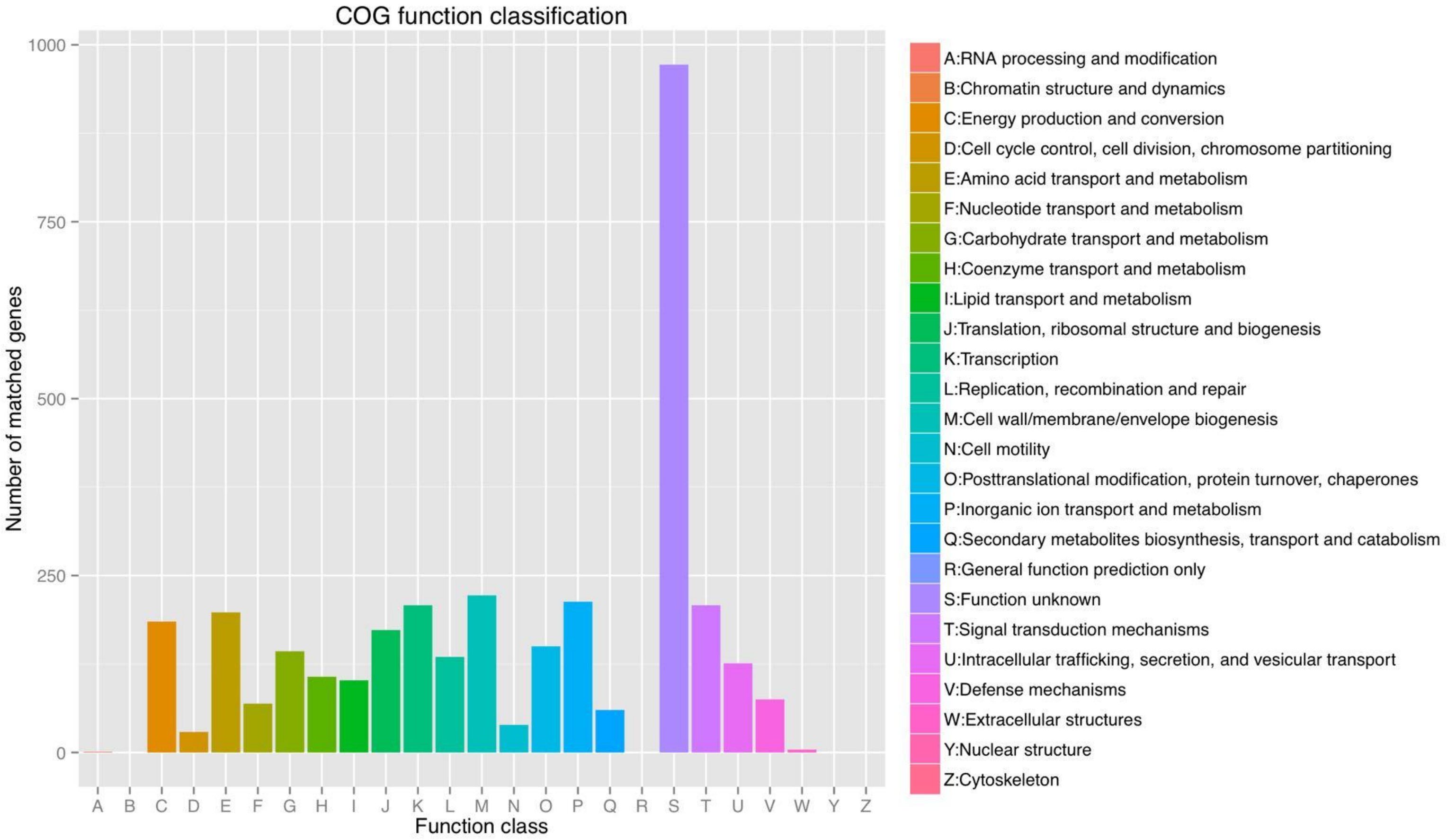

Additional file 12

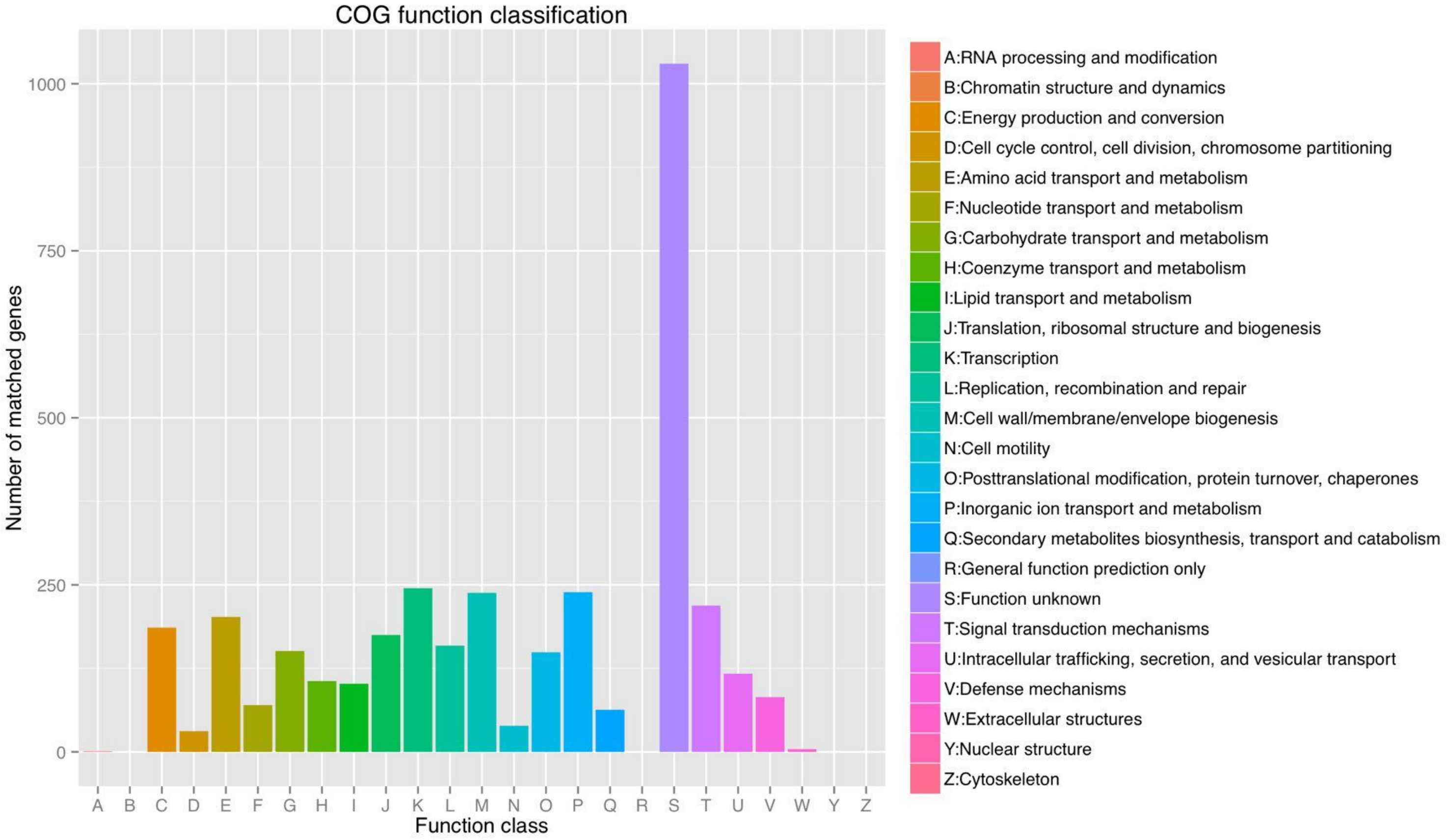

Additional file 13

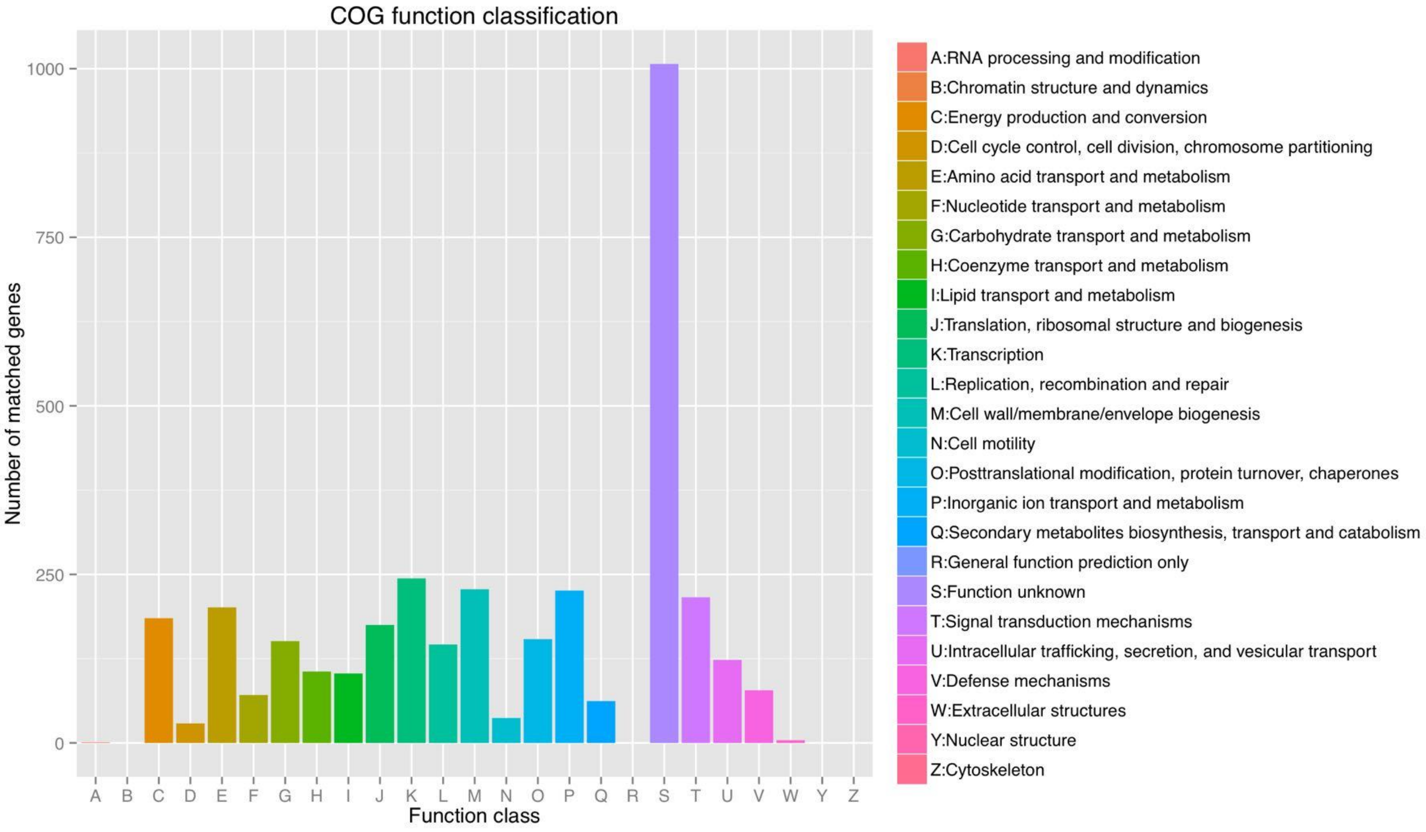

Additional file 14

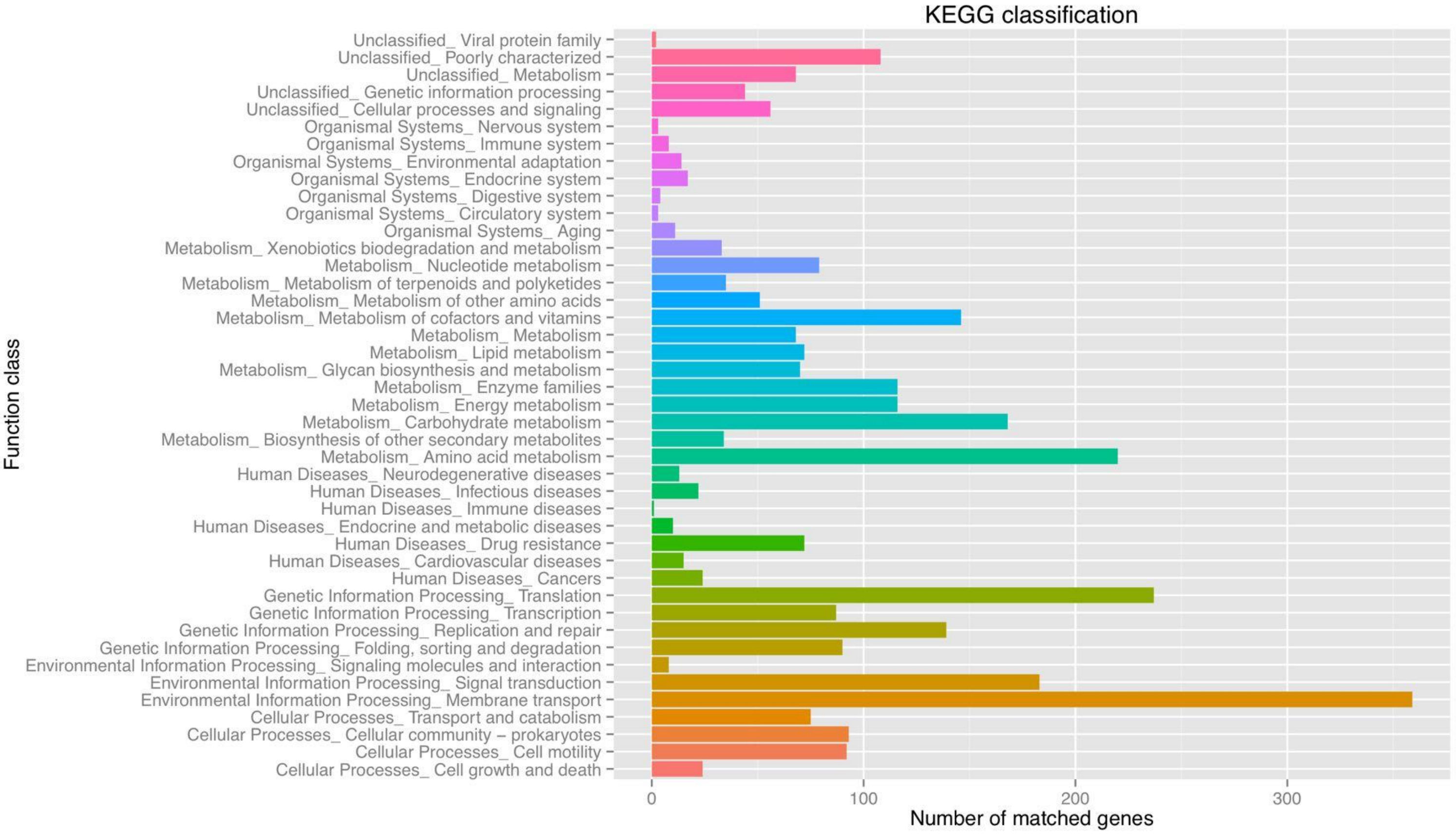

Additional file 15

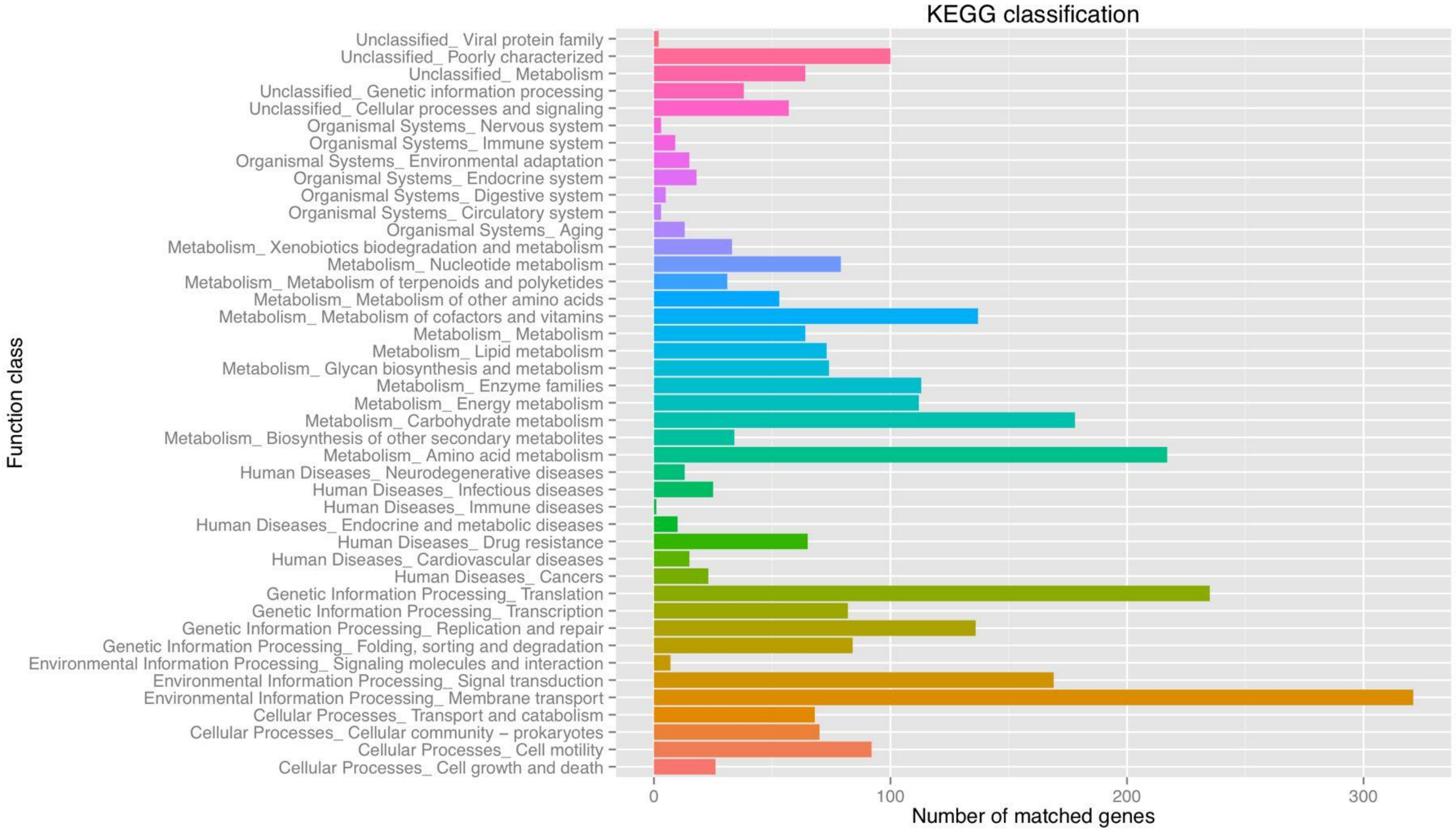

Additional file 16

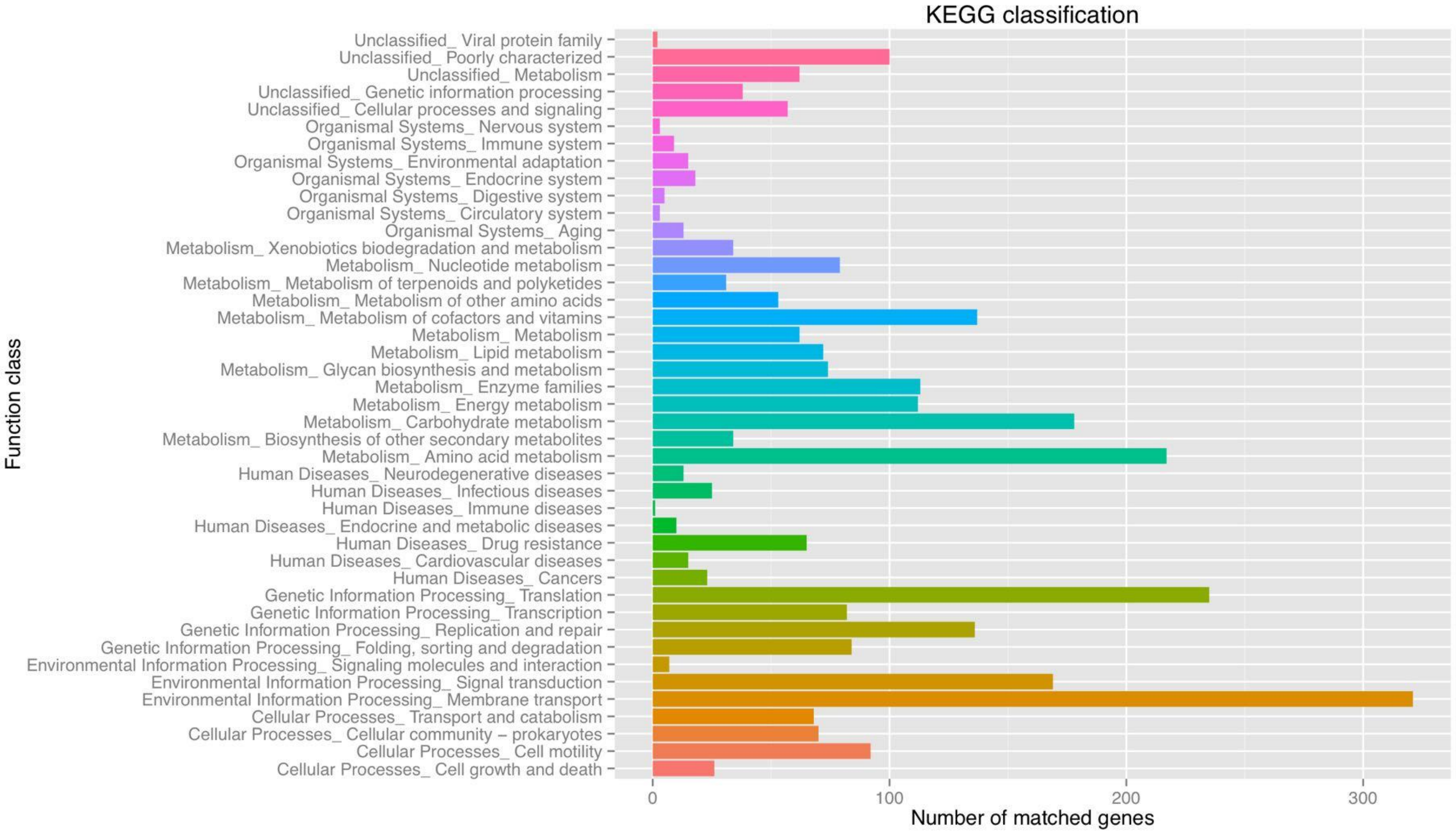

Additional file 17

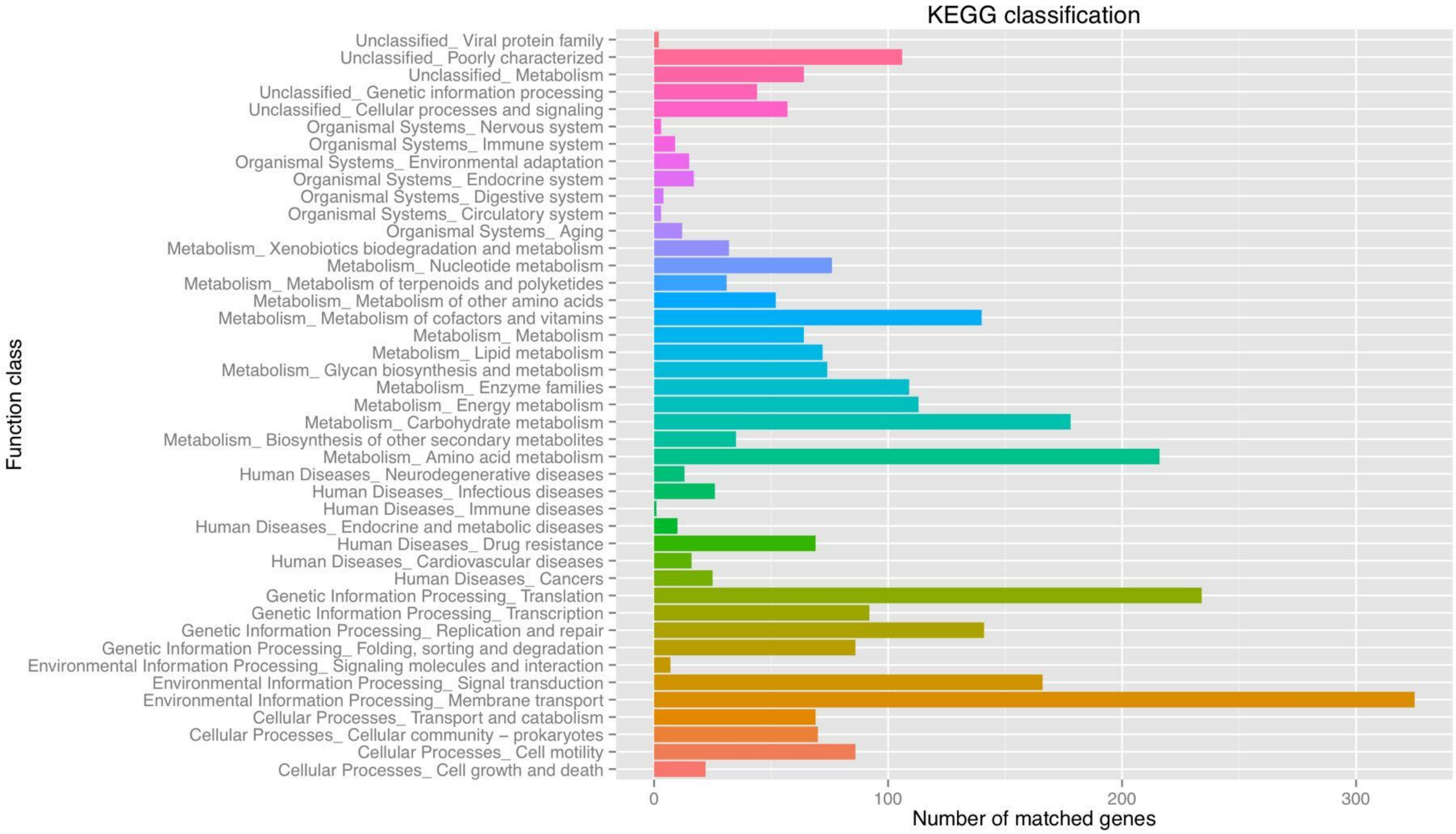

Additional file 18

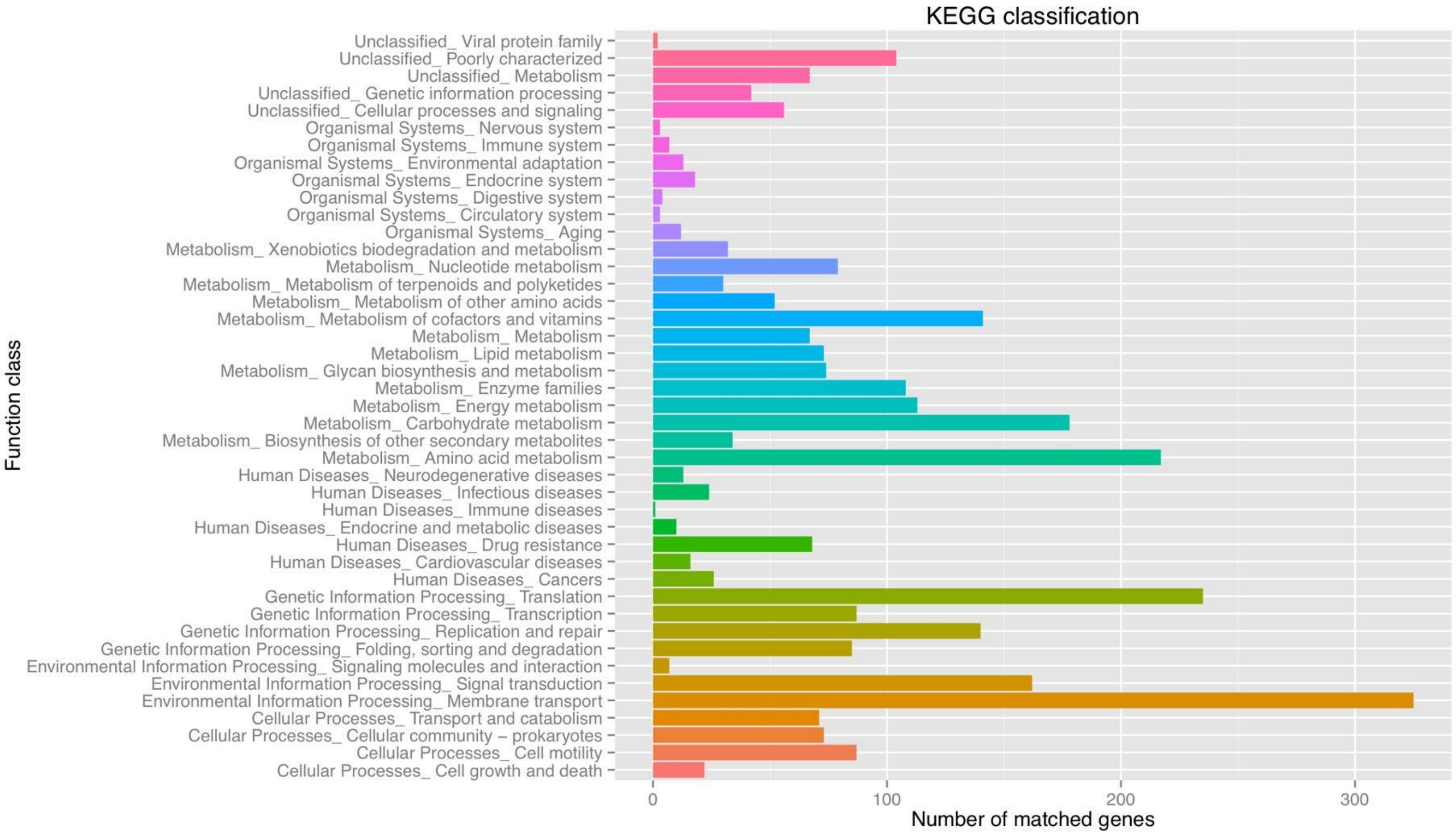

Additional file 19

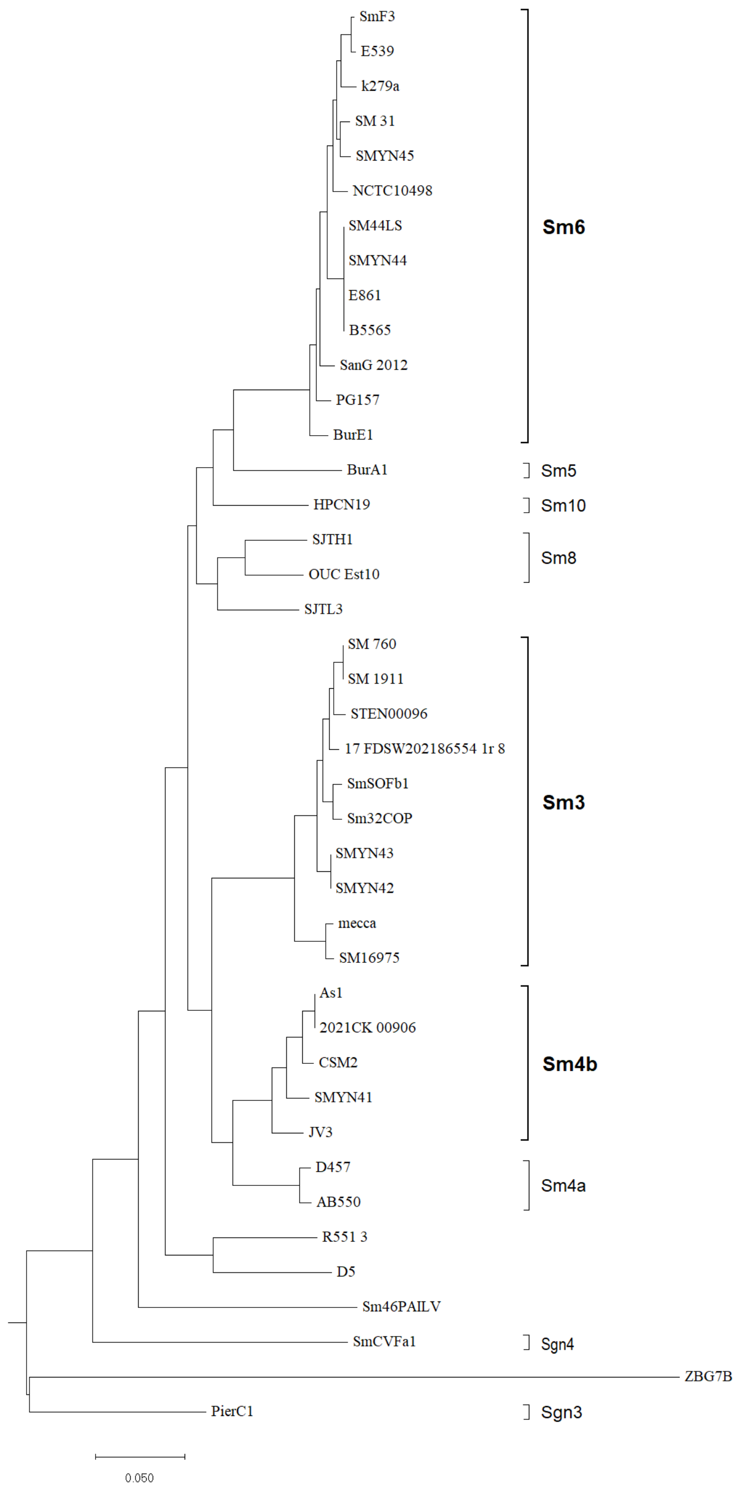

Additional file 20. Alleles and sequence types (STs) of the 30 *Stenotrophomonas maltophilia* strains according to the PubMLST.org website

Alleles	**atpD**	**gapA**	**guaA**	**mutM**	**nuoD**	**ppsA**	**recA**	***ST***
17_FDSW202186554-1r-8	13	172	399	50	121	121	22	564
2021CK-00906	217	111	606	104	135	122	147	997
AB550	2	2	163	2	2	3	5	219
B5565	3	4	24	7	7	22	7	31
BurA1	205	171	571	275	72	300	166	939
BurE1	125	265	565	6	8	296	7	929
CSM2	118	142	211	104	135	157	129	338
D457	2	122	556	269	213	292	128	930
D5.1	203	266	566	273	216	297	259	931
E539	1	1	307	6	28	4	6	996
E861	3	4	24	7	7	22	7	31
HPCN19	89	70	568	272	215	223	201	932
JV3	13	269	572	276	218	298	261	936
K279a	1	1	1	1	1	1	1	1
mecca	72	77	94	60	71	85	67	121
NCTC10498	3	1	1	3	6	4	1	27
OUC_EST10	193	267	567	278	214	302	258	940
PG157	5	83	104	51	79	80	73	131
PierC1	204	268	569	274	217	299	260	942
R551-3	35	36	44	37	37	39	32	45
SanG_2012	5	22	9	4	27	5	7	5
SJTH1	85	85	573	77	183	301	75	937
SJTL3	6	18	570	18	21	9	20	933
SM16975	72	77	321	166	71	85	67	934
SM-1911	13	69	168	50	230	230	22	998
SM-31	3	1	604	1	6	37	1	999
Sm32COP	147	27	574	277	155	29	22	938
SM44LS	3	4	24	7	7	22	7	31
SM-760	13	69	168	50	230	230	22	998
SmCVFa1	14	270	87	89	219	284	262	941
SmF3	1	1	307	6	1	4	6	935
SMYN41	101	264	563	271	211	294	157	925
SMYN42	13	28	564	116	212	143	22	926
SMYN43	13	28	564	116	212	143	22	926
SMYN44	3	4	24	7	7	22	7	31
SMYN45	1	1	326	3	25	295	1	928
STEN00096	13	69	265	91	112	121	22	356

Additional file 21

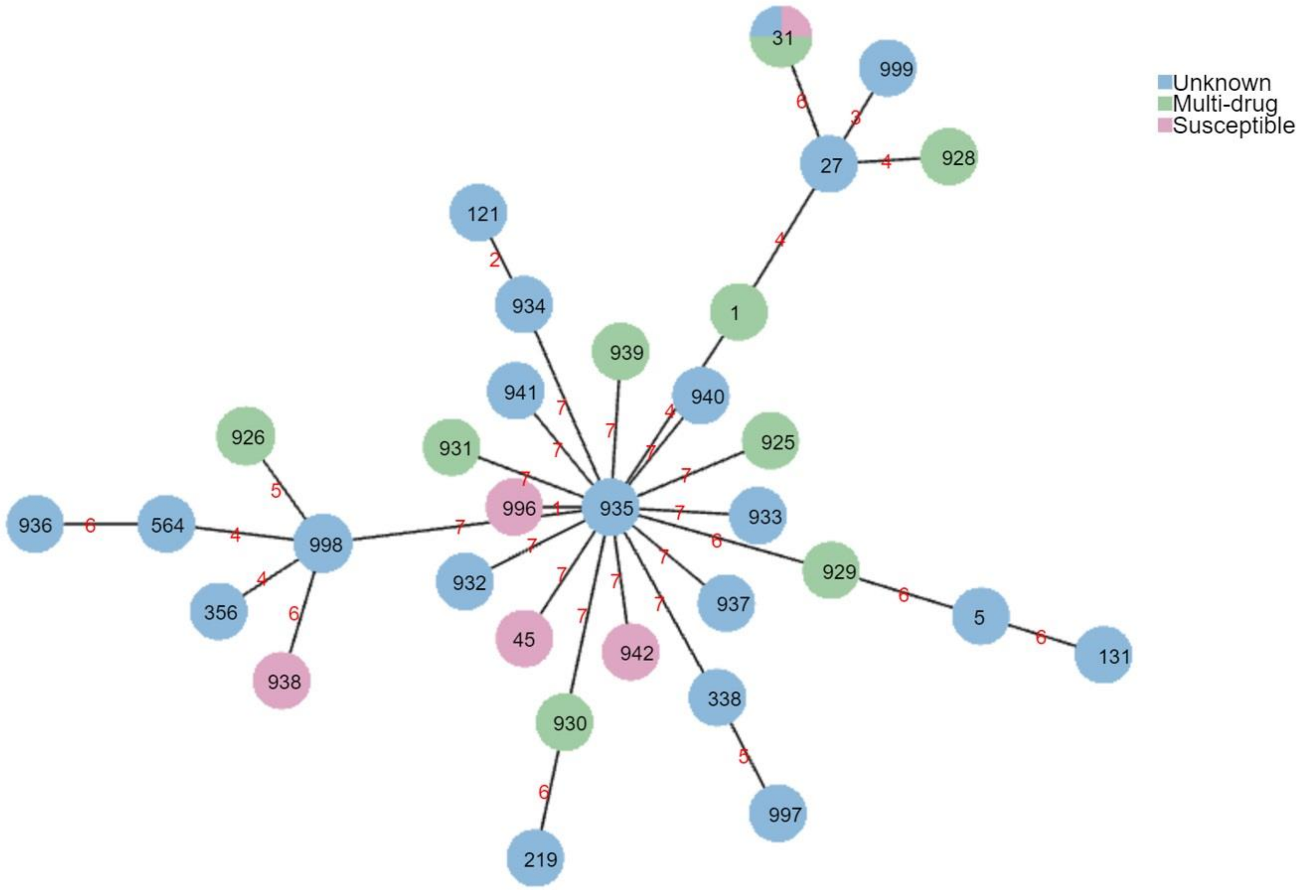

Additional file 22

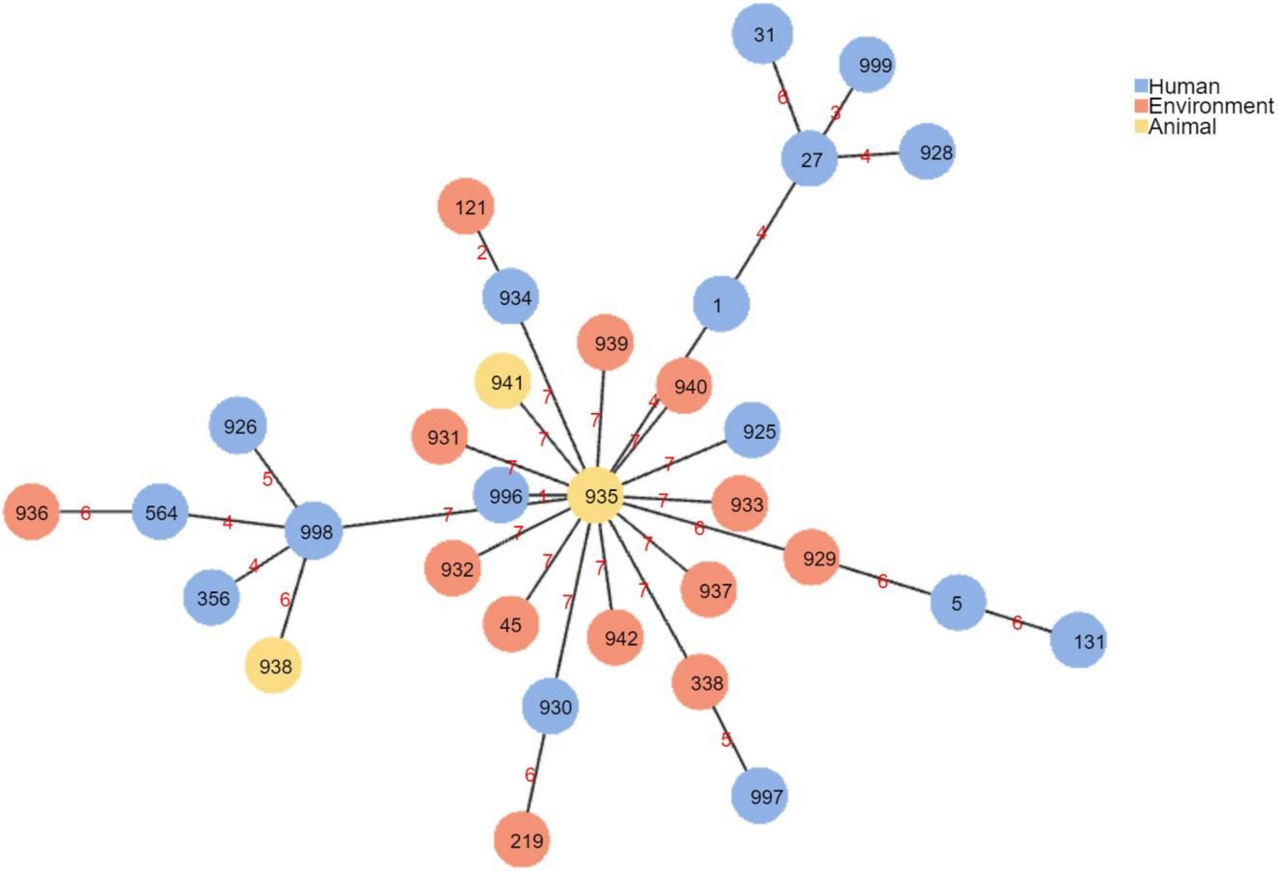

Supplement: Supplementary file 1 [file DataSheet_1.pdf]
